# Redox regulation: mechanisms, biology and therapeutic targets in diseases

**DOI:** 10.1038/s41392-024-02095-6

**Published:** 2025-03-07

**Authors:** Bowen Li, Hui Ming, Siyuan Qin, Edouard C. Nice, Jingsi Dong, Zhongyan Du, Canhua Huang

**Affiliations:** 1 Department of Biotherapy, Institute of Oxidative Stress Medicine, Cancer Center and State Key Laboratory of Biotherapy, West China Hospital and West China School of Basic Medical Sciences and Forensic Medicine, Sichuan University, Chengdu, PR China; 2 Frontiers Medical Center, Tianfu Jincheng Laboratory, Chengdu, PR China; 3 Department of Biochemistry and Molecular Biology, Monash University, Clayton, VIC Australia; 4 Department of Thoracic Surgery, West China Hospital, Sichuan University, Chengdu, Sichuan China; 5 Lung Cancer Center/Lung Cancer Institute, West China Hospital, Sichuan University, Chengdu, Sichuan China; 6 School of Basic Medical Sciences, Zhejiang Chinese Medical University, Hangzhou, China; 7 Key Laboratory of Blood-stasis-toxin Syndrome of Zhejiang Province, Hangzhou, China

**Keywords:** Senescence, Molecular medicine

## Abstract

Redox signaling acts as a critical mediator in the dynamic interactions between organisms and their external environment, profoundly influencing both the onset and progression of various diseases. Under physiological conditions, oxidative free radicals generated by the mitochondrial oxidative respiratory chain, endoplasmic reticulum, and NADPH oxidases can be effectively neutralized by NRF2-mediated antioxidant responses. These responses elevate the synthesis of superoxide dismutase (SOD), catalase, as well as key molecules like nicotinamide adenine dinucleotide phosphate (NADPH) and glutathione (GSH), thereby maintaining cellular redox homeostasis. Disruption of this finely tuned equilibrium is closely linked to the pathogenesis of a wide range of diseases. Recent advances have broadened our understanding of the molecular mechanisms underpinning this dysregulation, highlighting the pivotal roles of genomic instability, epigenetic modifications, protein degradation, and metabolic reprogramming. These findings provide a foundation for exploring redox regulation as a mechanistic basis for improving therapeutic strategies. While antioxidant-based therapies have shown early promise in conditions where oxidative stress plays a primary pathological role, their efficacy in diseases characterized by complex, multifactorial etiologies remains controversial. A deeper, context-specific understanding of redox signaling, particularly the roles of redox-sensitive proteins, is critical for designing targeted therapies aimed at re-establishing redox balance. Emerging small molecule inhibitors that target specific cysteine residues in redox-sensitive proteins have demonstrated promising preclinical outcomes, setting the stage for forthcoming clinical trials. In this review, we summarize our current understanding of the intricate relationship between oxidative stress and disease pathogenesis and also discuss how these insights can be leveraged to optimize therapeutic strategies in clinical practice.

## Introduction

The term “redox” is a portmanteau derived from “reduction” and “oxidation,” describing the chemical process involving the transfer of electrons between reactants in chemical reactions.^1^ Redox reactions are fundamental reactions in chemistry, occurring not only ubiquitously in everyday life but also alongside biological activities.^2,3^ The acquisition of energy in organisms is closely associated with redox reactions, where the energy supply originates from oxidative respiration within cells.^4^ For example, in the mitochondrial respiratory chain, the electron transfer process involves sequential redox reactions accompanied by the gradual release of energy.^5,6^ During this process, known as oxidative phosphorylation, energy drives the phosphorylation of ADP to produce ATP, providing energy for various activities.^7,8^ During the course of redox reactions, a series of reactive oxygen species (ROS) with oxidizing capabilities can be produced within organisms, including superoxide, singlet oxygen, hydrogen peroxide, and hydroxyl radicals.^9^ Hydrogen peroxide was synthesized in 1818, marking the first known synthesis of ROS. Radicals were not confirmed in living organisms until 1954.^10^ In 1967, it was found that the mitochondrial electron transport chain could produce hydrogen peroxide.^11^ Further research identified the NADPH oxidase (NOX) system as a critical source of H_2_O_2_ in cells, including non-phagocytic ones, emphasizing its signaling role under physiological conditions.^12^ Now we recognize that ROS can be generated through mechanisms such as the mitochondrial electron transport chain, the endoplasmic reticulum, and NOX.^13,14^

ROS can be scavenged by various reducing substances, including reducing small molecules, redox enzymes, and high-abundance redox proteins.^15,16^ The antioxidative system in organisms possesses a sensing mechanism for oxidants or electrophilic agents, which activates the transcription of antioxidative genes by triggering relevant transcription factors, thereby promoting the expression of antioxidative enzymes.^17^ Despite the presence of several antioxidative transcription factors in organisms, including AP-1^18^ and HO-1,^19^ NRF2 is often referred to as the master regulator.^20^ NRF2 was first identified in 1994,^21^ and by 1996,^22^ it had been discovered that under oxidative stress conditions, it could activate the expression of antioxidative enzyme genes, including NQO1, GPX4, TXN, and PRDX1.^23^ Due to the superior efficacy of antioxidative enzymes in scavenging ROS compared to exogenous molecules, the antioxidative effects mediated by NRF2 are notably significant.^24^ Additionally, based on their importance, the antioxidative enzymes in the body can be categorized into the first and second lines of defense against oxidative stress.^25^ The first line includes superoxide dismutase (SOD), catalase, and glutathione peroxidase (GPx), which encompass cytosolic SOD1 and mitochondrial matrix SOD2 that remove O_2_^•−^, catalases, and GPxs that eliminate H_2_O_2_, and certain GPxs and PRDXs that reduce lipid peroxides.^26^ By 1969, Researchers have discovered that SOD exhibits physiological catalytic activity by reducing O_2_^•−^ to hydrogen peroxide, which was the first antioxidant enzyme discovered to utilize superoxide anions as substrates.^27^ Consequently, many current antioxidant strategies are designed around SOD. The second line of defense involves the use of NADPH to reduce GSSG and TrxS2 by TRX, GCL, and glutathione synthetase responsible for GSH synthesis, glutathione reductase, and thioredoxin reductase.^28^ In typical organisms, the generation and clearance of ROS are maintained in a balanced state (redox homeostasis).^29,30^ In 1985, oxidative stress was defined as a cellular imbalance between oxidants and reductants, leading to the differentiation of eustress and distress to describe oxidative stress states under physiological and pathological conditions, thereby advancing our understanding of oxidative stress in human diseases.^31,32^ The traditional view is that ROS are toxic byproducts of body metabolism, destroying macromolecules and leading to pathogenic processes.^33,34^ However, with the increasing understanding of redox reactions, multiple lines of experimental evidence show that redox reactions are similar to many other modification modes and can affect molecular signaling pathways through redox modification, thereby affecting various biological activities.^35–37^

Thiols, highly reactive constituents in protein residues, serve as crucial agents in the transduction of redox signals and their interactions with small molecules.^38,39^ Influenced by ROS, thiols can participate in reversible oxidative reactions, including the formation of disulfide bonds (S-S), S-glutathionylation (SSG), S-nitrosylation (SNO), and S-sulfenylation (SOH).^40,41^ These oxidative modifications of cysteine can be reverted to the free thiol state (-SH) by specific reductants.^42,43^ These redox alterations are instrumental in modulating protein structure and functionality, subsequently affecting the cellular physiological processes.^44^ The principles of the Redox Code were raised in 2015,^45^ including the regulation of NADH and NADPH systems in metabolism, dynamic control of thiol switches in the redox proteome, activation and deactivation cycles of H_2_O_2_ production, and the response of redox signaling to environmental changes at various cellular levels, paved the way for new insights into disease-specific therapeutic targets through advanced biotechnological exploration of redox proteomics. In addition to directly affecting genome stability, redox signaling can also affect biological processes through nongenetic pathways, including epigenetic modification^46,47^ and protein homeostasis.^48^ Investigating the mechanisms of redox regulation can provide novel strategies for the treatment of human diseases from a new perspective.

Redox regulation is crucial in multiple human diseases characterized primarily by two mechanisms.^24^ The accumulation of ROS in cells directly damages biomolecules such as nucleic acids, membrane lipids, structural proteins, and enzymes, leading to cellular dysfunction or death.^49,50^ Alternatively, dysregulation in redox modifications leads to aberrant redox signaling, in which hydrogen peroxide generated under physiological or pathological stimuli serves as a secondary messenger, closely linked to the cellular redox state.^51^ Diseases like atherosclerosis, radiation-induced lung injury, and paraquat poisoning are directly attributed to or primarily caused by redox imbalances.^52–54^ In contrast, in conditions such as chronic obstructive pulmonary disease,^55^ idiopathic pulmonary fibrosis,^56^ hypertension,^57^ type II diabetes,^58^ neurodegenerative diseases,^59^ cancer,^13^ and systemic inflammatory response syndrome,^60^ redox regulation indirectly influences disease progression through signal transduction pathways. In these scenarios, redox signaling pathways do not regulate biomolecular and cellular activities through direct oxidative damage, but rather through redox modifications and the downstream signaling pathways they activate.^61^ In such cases, redox signaling regulation intersect with various cellular molecular events and is subject to reversible control, a biological function often overlooked in previous scientific research.^62^ These molecular events typically include those related to stress responses within the cell, such as DNA repair, epigenetic regulation, protein homeostasis, metabolic regulation, and the modulation of the extracellular microenvironment.^63,64^ Ultimately, through regulation at the tissue and organ levels, these processes influence the onset and progression of diseases in the organism. Antioxidant treatments may mitigate or treat diseases where redox imbalance is a primary factor.^65–67^ However, given the complexity of redox signaling, broad-spectrum antioxidant interventions can lead to adverse effects and may not fulfill therapeutic objectives.^68^ Therefore, understanding redox regulation mechanisms in diverse human diseases and identifying specific drug targets or critical modification sites may provide novel approaches for devising precise therapies for intractable diseases.

**Fig. 1:**
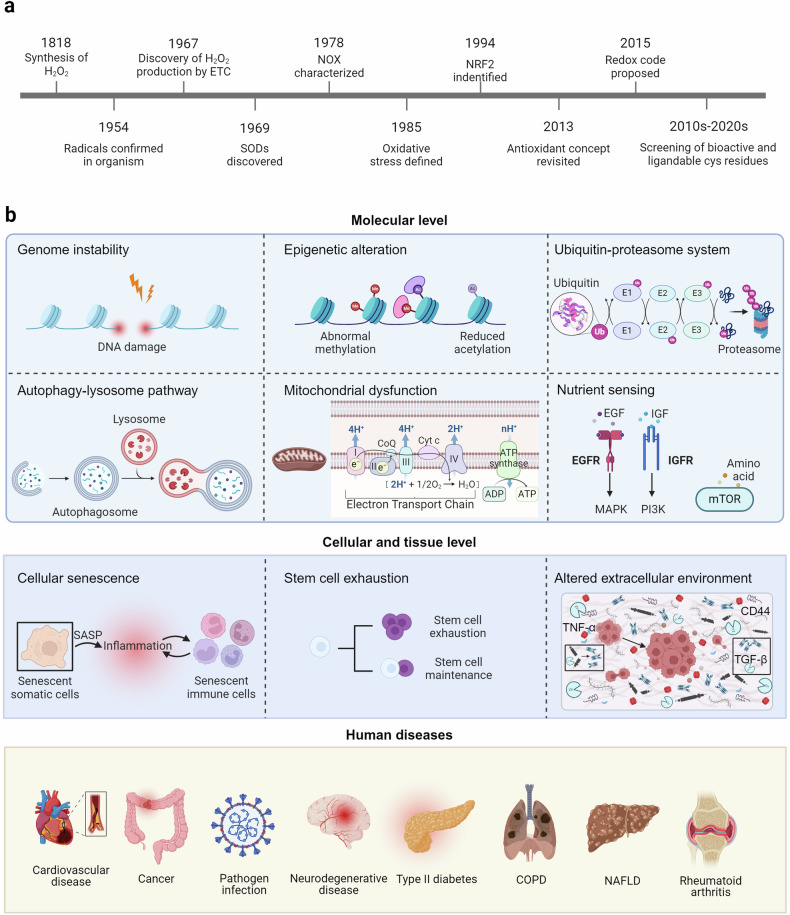
The brief history of redox signaling and its regulatory mechanisms in diseases. **a** Brief history of redox signaling research. **b** Redox signaling regulates human diseases in molecular, cellular, and tissue levels. The molecular level included genome instability, epigenetic alteration, dysfunction of the ubiquitin-proteasome system and autophagy-lysosome pathway, mitochondrial dysfunction, and nutrient sensing. The cellular and tissue level contains cellular senescence, stem cell exhaustion, and altered extracellular environment

This review will focus on the intricate relationship between oxidative stress and human diseases, elucidating the molecular mechanisms underlying the impact of redox signaling (Fig. 1). Additionally, we summarize the current potential redox regulatory targets and the latest clinical research progress, which are expected to provide new strategies for treating human diseases.

## Redox signaling orchestrated regulation of genomic stability

**Fig. 2:**
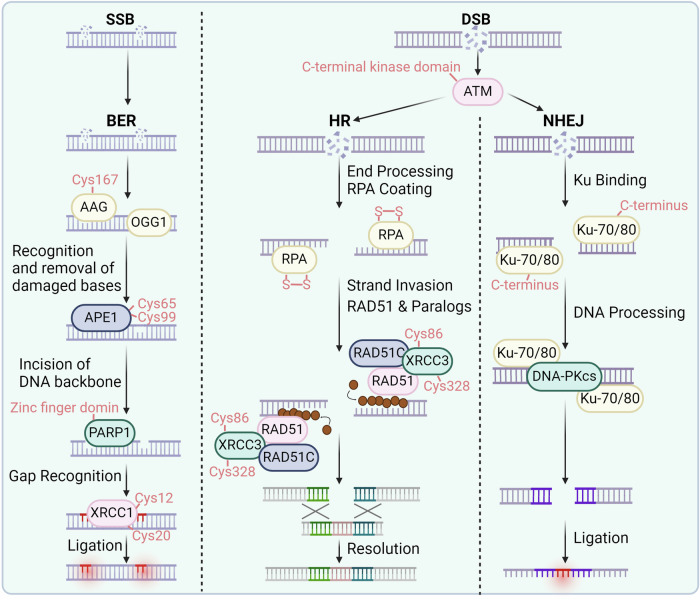
Redox signaling regulates DNA repair. In the DNA double-strand break process, the essential sensor ATM can be oxidized at the C-terminal kinase domain (Cys2991). In HR, RPA, XRCC3, and RAD51 can be oxidized at the indicated cysteines. In NHEJ, KU70/80 and DNA-PKcs can be oxidized and regulated. In BER process, AAG, OGG1, APE1, PARP, and XRCC1 can be oxidized at the specific cysteines

The genome within organisms and cells plays a critical role in health and disease. Disruption of its integrity is closely associated with the onset of various diseases, including cancer, neurological abnormalities, immune deficiencies, and aging.^69–71^ During life, organisms commonly face various sources of stress from the external environment and internal cellular factors.^72^ Among these factors, oxidative stress is one of the significant factors contributing to the disruption of genome integrity and stability.^73^ External factors such as psychological stress, radiation, and mechanical pressure can increase the levels of oxidative stress within cells.^9^ This in turn promotes the accumulation of reactive oxygen species.^74,75^ During DNA replication or transcription, ROS can chemically induce DNA missense mutations, truncation mutations, or even DNA breakage.^76,77^ Consequently, the integrity of the DNA is compromised, ultimately leading to the destabilization of proteins and RNA within cells and promoting the pathological process.^78^ Apart from its significant impact on DNA damage, redox signaling can also influence the repair status of DNA damage by finely regulating the redox modifications of DNA repair-related proteins (Fig. 2).^79,80^ Therefore, in-depth research summarizing the detailed regulatory effects of redox signaling on DNA repair proteins will contribute to understanding and developing clinical intervention measures.

### Double-strand breaks

DNA double-strand breaks (DSBs) are common and significantly impactful forms of DNA damage that typically occur when cells are exposed to ionizing radiation or during replication.^81^ DNA double-strand damage and defects in its repair mechanisms are associated with radiation sensitivity, aging, tumorigenesis, as well as neurological, immunological, and developmental deficiencies.^82,83^ These repair mechanisms require the involvement and catalysis of multiple essential proteins, many of which are finely regulated by redox signaling. The activation of ataxia-telangiectasia mutated protein kinase (ATM) is triggered by the Mre11-Rad50-Nbs1 (MRN) complex when damage occurs, facilitating the repair of double-strand breaks.^84,85^ Upon activation, the C-terminal kinase domain of ATM undergoes autophosphorylation, attracting DNA repair proteins such as p53 and CHK2 to regulate the cell cycle.^86,87^ Dysfunction of these proteins can result in genomic instability and Ataxia-Telangiectasia syndrome.^88–90^ Oxidative stress can modify ATM through cysteine oxidation, phosphorylation, and acetylation,^91^ which leads to ATM activation and subsequent phosphorylation of downstream proteins independent of MRN.^92^ Active ATM forms dimers via disulfide bonds, particularly at Cys2991. Mutation at Cys2991 abolishes redox sensitivity while maintaining activation by DNA damage.^93^ Similarly, a truncation mutation (R3047X) located near Cys2991 in ATM results in insensitivity to oxidative stress.^94^ The role of ATM as a sensor of oxidative stress suggests a novel function independent of DNA damage repair that is crucial for preventing DNA damage under oxidative stress conditions.

Typically, cells have two options for fixing DSBs: homologous recombination and nonhomologous end joining.^95,96^ Homologous recombination is a precise repair process mainly active in the S and G2 stages of the cell cycle, utilizing a sister chromatid as a repair template.^97,98^ DSBs are first detected, then the DNA ends are trimmed to form 3′-ssDNA. Replication protein A (RPA) stabilizes single-stranded DNA (ssDNA), which is subsequently substituted by RAD51 to create a nucleoprotein filament crucial for the processes of homology searching and strand invasion.^99,100^ The RAD51-ssDNA filament penetrates a similar DNA sequence, typically the sister chromatid, creating a displacement loop (D-loop) and initiating the repair process guided by a template. The oxidation-reduction state of the RAD51 Cys319 site regulates the formation of RAD51 foci. Upon exposure to DNA damage-inducing stimuli such as ionizing radiation, the RAD51 Cys319 site undergoes oxidation, allowing it to polymerize with PRDX1, thereby facilitating the formation of RAD51 foci.^101^ This process recruits DNA damage repair proteins to sites of DNA damage, thereby promoting DNA repair.^102–104^ However, in the absence of PRDX1, the formation of RAD51 foci is impaired.^105^ Moreover, XRCC3 plays a crucial role in homologous recombination repair via RAD51.^106^ Its absence disrupts RAD51 focus formation, inhibiting recombination repair and causing genomic instability.^107^ The sensitivity of XRCC3 to UVA-induced oxidative stress is associated with structural changes, possibly due to C-terminal oxidation blocking antibody detection regions. This oxidation sensitivity is reversible (Cys86, Cys328); mutating all cysteines to serine abolishes this effect.^108^ Additionally, XRCC3 oxidation affects its response to DNA damage,^109^ indicating that the oxidation state may influence its role in oxidative stress versus DNA repair.

On the other hand, nonhomologous end joining is a repair process that is prone to errors and does not require a matching template.^110,111^ Nonhomologous end joining plays a vital role in the repair of double-strand breaks, particularly in cells that are not actively replicating, by directly connecting the damaged ends of DNA.^112–114^ Initiating the procedure involves attaching the Ku70/Ku80 heterodimer to the ends of DNA, shielding them from over-resection.^115,116^ This complex recruits other proteins, including DNA-PKcs, which form a bridge over the DSB to facilitate end processing.^117^ End-processing enzymes, such as Artemis, trim DNA ends to prepare them for direct ligation.^118,119^ Finally, the ligase IV complex, which includes XRCC4, XLF, and PAXX, ligates the processed ends together, albeit sometimes incorporating small deletions or insertions at the junction site.^120,121^

Ku forms a DNA-end ring, facilitating end rejoining, with high DNA binding affinity and unknown dissociation mechanisms.^122,123^ Oxidative stress from UV exposure inhibits Ku-DNA binding, which can be reversible by reducing agents.^124,125^ Oxidation induces conformational changes, enhancing dissociation rates, particularly in the C-terminus of Ku80, which contains Cys493 and Cys638 and is crucial for DNA-PKcs recruitment.^126^ The redox state of Ku influences DNA binding and downstream protein recruitment, suggesting that oxidation may regulate repair pathway selection, aiding in repair pathway decisions. DNA-PKcs, a critical component in the repair of double-strand breaks through NHEJ, plays a crucial role in recognizing DNA damage and facilitating DNA repair.^127,128^ With 4128 amino acids (87 Cys), DNA-PKcs is sensitive to oxidative stress and nitrosylation, impacting its activity and expression.^129,130^ Nitric oxide enhances DNA-PKcs expression and activity via SP1 binding, potentially protecting against DNA damage.^131^ The production of RONS increases the sensitivity of cells to genotoxic drugs by decreasing the activity of DNA-PKcs, hindering repair processes, and leading to the accumulation of DSBs.

In summary, contrary to traditional views that oxidative stress merely leads to increased DNA damage and disease progression, a deeper understanding of redox signaling reveals that redox modifications can enhance the activity of ATM, facilitating the formation of RAD51 foci. Additionally, redox modifications of Ku and DNA-PKcs each promote their biological functions, thereby aiding the repair of DNA double-strand breaks.

### Base excision repair

Base excision repair (BER) addresses base lesions, including oxidative and methylated damage, and maintains genomic stability.^132–134^ BER dysfunction leads to error propagation, DNA breaks, and genomic instability,^135,136^ leading to the progression of several diseases, including cancer, immune deficiency, and neurodegenerative diseases.^135,137^ Under oxidative stress, BER components are modified and inhibited, contributing to the accumulation of DNA damage.^138^ The BER process begins with the recognition and excision of the damaged base by DNA glycosylases.^139,140^ Alkyl-adenine DNA glycosylase (AAG) is a critical enzyme that removes methylated DNA bases in BER.^141,142^ Methyl methanesulfonate (MMS) treatment induces the formation of methylated DNA lesions via AAG.^143^ GSNO treatment enhances BER intermediates after MMS exposure, particularly during recovery, in an AAG-dependent manner. GSNO transfers an NO group to AAG, activating its activity by nitrosylating Cys167.^144^ Elevated GSNO inhibits downstream APE1, leading to base accumulation. Bases are mutagenic and can cause single-strand breaks and DSBs, exacerbating cell damage.

8-Oxoguanine glycosylase (OGG1) is crucial for recognizing and excising oxidized DNA bases, generating base excision repair.^145–147^ Research involving mice lacking OGG1 validated its ability to eliminate 8-oxoguanine (8oxoG) damage.^148,149^ OGG1 has eight Cys residues and is sensitive to oxidative stress induced by various oxidants, such as cadmium.^150,151^ Cadmium changes the redox status of cells by reducing glutathione levels and increasing reactive oxygen and nitrogen species, resulting in the inhibition of OGG1.^152,153^ This effect is not influenced by chelating agents but relies on antioxidants such as N-acetylcysteine (NAC).^154^ Nitrosylation also regulates OGG1, inhibiting its activity. Singlet oxygen from UV exposure also inhibits OGG1, causing oxidative DNA damage.^155^ The common OGG1 variant Ser326Cys is redox-sensitive due to introducing a Cys residue.^156^ Ser326Cys forms disulfide bonds under oxidative stress, leading to dimerization and nonproductive DNA binding, further inhibiting its activity.^157^ This variant also lacks tight DNA binding ability and coordination with BER steps, causing DNA damage accumulation and genomic instability, particularly under inflammatory conditions due to tumor necrosis factor-alpha (TNF-α)-induced oxidative stress and NO release.^158^ These findings underscore the link between inflammation, oxidative stress, and disease through OGG1 modulation.

In addition to OGG1, MUTYH is responsible for repairing oxidative DNA damage by removing adenine, which is incorrectly inserted across 8oxoG.^159,160^ MUTYH is a 60 kDa glycosylase that comprises 546 amino acids, including twelve Cys residues.^161^ Oxidative stress inhibits MUTYH activity, potentially impeding DNA damage repair and highlighting its susceptibility to redox modification.^162^ Thus, ROS generated by UV exposure can hinder two critical enzymes involved in oxidative DNA damage repair. APE1 consists of 318 amino acids, including seven Cys residues.^163,164^ It operates in BER and autonomously controls the oxidation‒reduction status of transcription factors.^165,166^ The N-terminus of APE1 facilitates protein‒protein interactions and redox activity, while the C-terminus mediates endonuclease activity.^167^ Redox sensitivity involves Cys65, which is essential for the biological function of APE1.^168^ Oxidative modifications, such as nitrosylation at Cys93 and Cys310, influence subcellular localization.^169^ Oxidative stress induces reversible glutathionylation at Cys99, inhibiting DNA binding and endonuclease activity of APE1.^170^

PARP1 contains 3 zinc finger domains crucial for DNA‒protein interactions, shielding Cys residues from oxidation.^171,172^ Heavy metals such as arsenic displace zinc, heightening Cys sensitivity to oxidative stress and inhibiting DNA binding.^173^ Nitrosylation disrupts zinc coordination, inhibiting PARP1 and leading to DNA damage accumulation.^174^ XRCC1, a protein of 69.5 kilodaltons and consists of 633 amino acids (including six cysteines), aids in recruiting proteins for base excision repair by interacting with PARP1, ligase III, APE1, and polβ.^175^ Its N-terminal domain, which contains redox-sensitive Cys residues (Cys12, Cys20), binds polβ. The oxidation process causes a 6.4 Å shift between cysteine 12 and cysteine 20, resulting in a 25-fold increase in the attraction of XRCC1 to polβ.^176^ Cys12 is crucial for polβ recruitment; mutating it to Cys12Ala delays recruitment.^177^ Pro2 forms a stabilizing proline carbamate adduct upon CO2 exposure. The efficiency of base excision repair and DNA repair is influenced by the conformational changes caused by oxidation in XRCC1.

Unlike double-strand DNA repair, redox modifications play a dual role. Redox modifications enhance the enzymatic activity of AAG and promote the interaction between XRCC1 and polβ, facilitating DNA repair. However, the enzymatic activities of OGG1, MUTYH, and APE1 are inhibited following redox modifications. These varied outcomes resulting from redox modifications reflect the diversity in biological functions of different redox modifications and modifications at specific cysteine sites on various proteins. Therefore, comprehensive consideration of the overall outcomes should be given when developing therapeutic models that regulate global redox states.

### Direct reversal pathway

The direct reversal pathway of DNA repair is a critical and efficient mechanism that directly corrects certain types of DNA damage, restoring the original DNA structure without requiring extensive processing or synthesis.^178–180^ One of the most well-characterized reactions in this pathway is the repair of methylated guanine.^181,182^ When guanine bases in DNA are alkylated to form O^6^-methylguanine, they can mispair with thymine during replication, leading to mutations.^183,184^ O^6^ methylguanine methyltransferase (MGMT) is vital for repairing O^6^ methylguanine lesions and prevents GC-to-AT mutations.^185^ MGMT, comprising 207 amino acids with five Cys residues and a molecular weight of 21 kDa, transfers methyl groups to Cys145, its active site.^186^ This action triggers MGMT degradation, making it a suicide protein. Nitrosylation of Cys145 by nitrosoglutathione (GSNO) inactivates MGMT, leading to its ubiquitination and proteasomal degradation and reducing its half-life from 24 to 1.3 h.^187^ This degradation results in DNA damage, potentially contributing to hepatocarcinogenesis, particularly in hepatocellular tumors with high NO synthase and low GSNOR levels.

### Telomere attrition by oxidative stress

Telomeres are DNA‒protein complexes devoid of gene coding function located at the ends of cell chromosomes.^188,189^ As cells replicate their DNA, the ends of chromosomes cannot be fully replicated, resulting in a loss of DNA at each cell division.^190,191^ Telomeres serve to protect the genomic integrity of chromosomes.^191^ Similar to DNA damage, telomeric sequences are also susceptible to oxidative stress-induced damage.^192^ While DNA damage often leads to genomic instability and tumorigenesis, damage at the telomeres can promote cell death.^193^ Numerous research papers have separately examined the topics of DNA damage and telomere shortening.^188^ Hypoxia-induced oxidative stress can be alleviated by human telomerase reverse transcriptase (hTERT) expression, which is facilitated by the presence of binding sites for hypoxia-inducible factor 1 (HIF-1) in the promoter of hTERT.^194,195^ Tankyrase (TNKS) is a crucial telomere-associated protein (TAP) that maintains the structural and functional integrity of telomeres. TNKS protects eroded telomeres from cellular senescence caused by ROS and ultraviolet radiation.^196^ Thus, TNKS appears crucial when external conditions damage telomeres.^197^ Telomeric damage may enhance TNKS activity, leading to the maintenance of telomere structural integrity beyond its usual level.^198^ Meanwhile, TNKS can also coordinate cell proliferation pauses by regulating p21 stability, thereby influencing the progression of various diseases, including cancer and aging.^199^ Even with various antioxidant systems in place, the production of ROS could surpass the ability of the defense network, leading to oxidative stress.^24^ Oxidative stress suppresses telomerase activity and promotes telomeric erosion.^200^ Telomeric DNA is prone to oxidative damage due to the less efficient repair of single-strand breaks caused by oxidative damage in telomeres compared to nontelomeric regions. Additionally, G-quadruplexes tend to accumulate 8oxoG lesions, which are affected by the base excision repair pathway.^201^ The DNA replication machinery is compromised by oxidative stress, leading to telomeric shortening as a result of decreased expression of hTERT.^202^ Hypoxia leads to the expression of HIFs, which control hTERT and modify the length of telomeres.^203^ The maintenance of telomeres is influenced by continuous exposure of cells to free radicals.

Overall, redox modifications generally promote DNA repair, while some DNA repair enzymes are inhibited by these modifications. These differences may arise from variations in oxidative stress levels in different experimental models, or due to the intrinsic properties of different DNA repair enzymes. Therefore, in exploring the regulation of redox states for disease treatment, precise targeting of specific DNA repair enzymes can be considered. This approach necessitates further research into the molecular mechanisms of redox signaling.

## Redox signaling-mediated epigenetic regulations

**Fig. 3:**
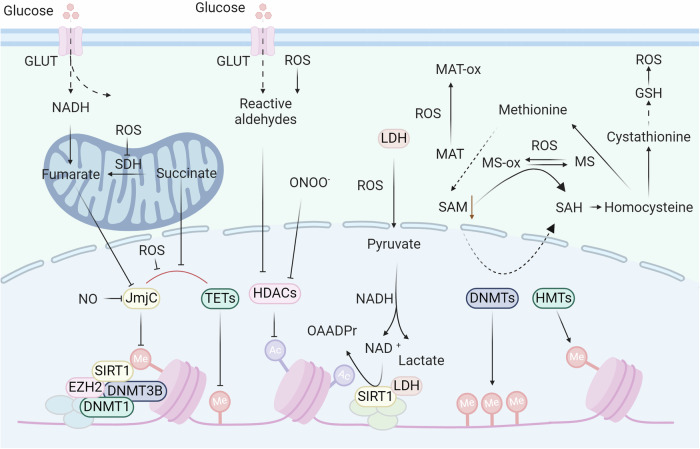
Epigenetic modifications are regulated by redox signaling. In the regulation of DNA and histone methylation, redox signaling can regulate the biosynthesis of SAM by oxidizing MAT. ROS can inhibit the enzyme activity of JmjC and TETs, thus regulating histone and DNA methylation. Additionally, redox signaling directly oxidizes DnaJb5 and HDACs to regulate histone acetylation

Epigenetics is a heritable and reversible change in gene expression that is not caused by gene sequence changes and involves regulating and maintaining gene expression.^204,205^ It has important implications for understanding biology and disease pathogenesis.^206,207^ Unlike diseases caused by alterations in genetic material, epigenetically mediated diseases are often induced by environmental factors.^208^ For instance, epigenetic modifications such as DNA methylation can influence gene expression within an organism without altering the DNA sequence and serve as a crucial mechanism in tumorigenesis.^209^ Moreover, psychiatric disorders including depression and schizophrenia, which are related to postnatal environmental factors, are also intricately linked with epigenetics.^210^ Additionally, epigenetics is associated with cardiovascular diseases,^211^ metabolic diseases,^212^ autoimmune disorders,^213^ endocrine disruptions,^214^ and aging.^215^ Recent studies have shown that redox regulates epigenetic modifications, including DNA methylation, histone modification, chromatin remodeling, and non-coding RNA (Fig. 3).^216,217^ Due to the reversible nature of epigenetics, identifying potential key epigenetic molecules affected by oxidative stress is expected to provide potential biomarkers and therapeutic targets for various human diseases.^218,219^

### DNA methylation

DNA methylation is one of the best-studied epigenetic modifications.^220–222^ DNA methyltransferase catalyzes specific DNA sequences, and S-adenosylmethionine is used as a methylation donor to achieve methylation group modification through covalent bonding.^223,224^ Recent research indicates that DNA methylation is possible on cytosine and adenine bases, including 5-methylcytosine (5mC), N^6^-methyladenine (6mA), and N^4^-methylcytosine (4mC).^225^ Among these modification types, the 5mC modification of CpG islands, which can repress gene transcription, is the most common DNA methylation in mammals.^226,227^ The level of DNA methylation in the body has the characteristics of an epigenetic clock, which shows that the overall level of epigenetic modifications in the body decreases with age. Furthermore, a study involving the sequencing of whole blood samples from 18,413 volunteers analyzed the association between DNA methylation and 19 diseases.^228^ Notably, type 2 diabetes, chronic obstructive pulmonary disease, chronic pain, breast cancer, and ischemic heart disease demonstrated strong associations with DNA methylation at specific loci. However, the underlying molecular mechanism needs to be further elucidated.^229,230^

Earlier studies on the effects of redox reactions on DNA focused on direct oxidative modification of DNA sequences.^231,232^ Cells exposed to hypochlorous acid (HOCl) exhibit heightened levels of chlorinated nucleobases (e.g., 5-chlorocytosine (5-ClC)).^233^ Further studies have shown that 5-ClC binds to the methyl-CpG-binding protein MeCP2 and disrupts the site specificity of DNA methyltransferases (DNMTs), resulting in changes in DNA methylation patterns.^234^ However, 5-ClC is detected at a relatively low frequency of 3 per 10^8^ bases, and its impact on mutagenicity may outweigh its effects on global DNA methylation.^235^ Additionally, the oxidation of guanosine to 8-oxo-2′-deoxyguanosine (8-oxodG) at CpG sites directly interferes with methylation.^236,237^ However, the reported extent of 8-oxodG occurrence is one lesion per 10^5^–10^6^ deoxyguanosines.^238^ Notably, mouse models^239^ of chronic inflammation and colonic samples from patients with inflammatory bowel disease (IBD)^240^ did not exhibit increased levels of 8-oxodG or other conventional oxidative DNA lesions compared to control samples. Therefore, recent research has focused on the biological significance of redox processing. Intriguingly, a recent study showed that exposure to sublethal hydrogen peroxide led to a long-term genome-wide decrease in DNA methylation.^241^ Another study used the neutrophil-derived oxidant glycine chloramine to treat Jurkat T lymphoma cells by inhibiting the activity of DNMT and the formation of the intracellular methylation donor SAM.^242–244^ Consistent with these findings, brain iron loading-mediated oxidative stress decreased the enzyme activity of DNMTs in mouse models, indicating an increase in the risk of neurodegeneration.^245^ In contrast to the biological functions described above, recent research has shown that hypoxia-mediated oxidative stress can increase the expression of DNMTs and facilitate hypermethylation of the NDGR2 promoter.^246^ These different results suggest that in-depth mechanistic research, such as the study of the effects of oxidative stress on DNMT enzyme activity, transcription, protein stability, and redox modification patterns, will help researchers understand the different biological functions of oxidative stress and develop new treatment strategies.

### Histone modification

The DNA nucleotide sequence winds around histones through electrostatic interactions in the chromosome. Modification of histones can affect the exposure of the DNA nucleotide sequence and thus affect the transcription level of the gene.^247–249^ Therefore, studying the regulatory mechanism of oxidative stress on histone modification is of great help in understanding the mechanism of selective protein expression.

#### Histone methylation

Histone methylation is one of the most common histone modifications.^250–252^ After specific amino acids (e.g., lysine or arginine) are methylated, they affect the tightness of DNA and nucleosome binding, affecting the transcription level of particular genes on DNA.^253^ The methylation of histones is a complicated process that controls the dynamics of chromatin and the transcription of genes. This balance is regulated by histone methyltransferases (HMTs) and histone demethylases (HDMs).^254^ Oxidative stress, such as H_2_O_2_ in diabetic retinopathy, increases H3K4me3 and H3K27me3 while reducing the acetylation of histones such as H3K9ac and H4K8ac.^255^ In diabetic nephropathy, 12(S)-HETE upregulates SET7, promoting profibrotic gene expression. Artery occlusion decreases H3K4me3 levels in astrocytes.^256^ PRMTs are oxidized by H_2_O_2_, reducing histone arginine methylation. LSD1 produces hydrogen peroxide in response to DNA damage. Base excision repair proteins influence epigenetic modifications.^257^ Additional investigations are needed to determine how redox agents, dosage, cell/tissue specificity, and pathological conditions affect epigenetic control and gene expression.

As mentioned earlier, DNMTs and HMTs add methyl groups to DNA or histones by transferring methyl groups from SAM to the substrate, resulting in the formation of S-adenosyl homocysteine (SAH).^258^ Methionine adenosyltransferase (MAT) converts methionine into SAM using ATP.^259^ The decrease in SAM levels caused by reactive oxygen/nitrogen species (RO/NS) results in a decrease in the activity of DNMTs and HMTs. Hydrogen peroxide causes a change in Cys121 of MAT by generating hydroxyl radicals, leading to the inactivation of MAT and a decrease in SAM biosynthesis.^260^ The SAM reduction induced by H_2_O_2_ in A549 cells leads to hypomethylation of long interspersed nuclear element-1 (LINE-1).^261^ Additional research is needed to clarify how various redox signals, dosage reactions, and short-term versus long-term exposure to RO/NS impact SAM, DNA, and histone alterations.

#### Histone acetylation

Histone acetylation is affected by RO/NS.^262–264^ At present, researchers have identified eighteen histone deacetylases (HDACs) that are classified as zinc/iron-dependent deacetylases, including class I (HDACs 1, 2, 3, and 8), class II (HDACs 4, 5, 7, and 9), class IIB (HDACs 6 and 10), and class IV (HDAC11).^265,266^ In contrast, sirtuins 1–7, class III HDACs, depend on NAD^+^ for their function and do not require zinc or iron.^267^ Numerous HDACs exhibit redox regulation. TRX1 reduces oxidized molecules, such as class II HDACs, through the thiol-disulfide exchange, which helps with nucleocytoplasmic shuttling.^268^ TRX1 induces DnaJb5, which forms a complex with class II HDACs. When exposed to ROS/H_2_O_2_, Cys274/Cys276 within DnaJb5 and Cys667/Cys669 in HDAC4 could undergo oxidation, leading to the creation of disulfide bonds within the molecules that can be reduced by TRX1. The importance of reducing Cys274/Cys276 in DnaJb5 for interaction with HDAC4 has been emphasized, as has the impact of decreasing Cys667/Cys669 in HDAC4 on preventing its nuclear export.^269^ Other Cys residues in HDACs are sensitive to H_2_O_2_. PRDXs act as cellular antioxidants and may relay H_2_O_2_ signaling, with PRDX I and II targeted by HDAC6, decreasing H_2_O_2_ reductive activity.^270^ Class I HDACs, notably HDAC1, are redox-sensitive, potentially via Cys residues, impacting histone acetylation patterns and gene expression.^271^ Cys102 and Cys153 in HDAC8 are integral to its redox switch, with disulfide bond formation under oxidative conditions potentially resulting in reversible enzyme activity loss.^272^ Overall, redox RO/NS modulates chromatin accessibility by altering HDAC/histone acetylation, necessitating further investigation into HDAC responses to redox signaling.

Histone acetylation is catalyzed by histone acetyltransferases (HATs).^273–275^ P300/CREB-binding protein (CBP) is one such protein.^276,277^ The enzymes alter preserved lysine (K) sites on histone tails by moving an acetyl group from acetyl-CoA to create ε-N-acetyl-lysine.^278^ This process causes a reduction in histone binding to DNA, leading to the relaxation of chromatin and increased transcription of genes.^279^ In *Escherichia coli*, an elevated ratio of NADH to NAD^+^ inhibits pyruvate dehydrogenase (PDH) activity, impeding the formation of acetyl-CoA, as the conversion of pyruvate to acetyl-CoA relies on PDH.^280^ This process causes a reduction in histone binding to DNA, leading to the relaxation of chromatin and increased gene transcription.^281^ In mammalian cells, H_2_O_2_ enhances HAT activity, resulting in increased acetylation of H3/H4 histones.

Redox modifications inhibit HDAC activity and may promote the activation of HAT, leading to an increase in histone acetylation and the promotion of gene expression. This process may be associated with the onset and progression of certain diseases, necessitating further experimental validation.

### Non-coding RNA

With the implementation of the Human Genome Project, researchers discovered that over 98% of the human genome consists of non-coding DNA.^282^ Further studies by the Encyclopedia of DNA Elements (ENCODE) project indicated that up to 80% of the human genome, including coding DNA, can undergo transcription, suggesting the presence of a substantial amount of non-coding RNA in the human body.^283^ Similar to DNA, oxidative stress can induce 8-oxoG modifications in RNA, potentially altering its stability or base pairing fidelity.^284^ Given the longer sequences of non-coding RNAs, the impact of 8-oxoG modifications might be less significant overall, whereas, for shorter microRNAs, these modifications could have a more pronounced effect. Researchers have examined the oxidation of nucleic acids in rat cardiac cells under oxidative stress conditions, with miR-184 exhibiting the most significant changes in oxidative modifications. Following oxidative modification, miR-184 acquires new nucleic acid pairing capabilities, allowing it to bind to the mRNA of BCL-xL and decrease its translation level, thereby promoting apoptosis.^285^ In the absence of oxidative modifications, this binding capability is absent, suggesting that redox modifications can specifically regulate the initiation of biological activities. Oxidative miRNA sequencing of a rat model with cardiac hypertrophy induced by oxidative stress revealed an 8-oxoG modification at position 7 of miR-1. Introduction of 7o8G-miR-1 alone could induce cardiac hypertrophy in mice, while specific inhibition of 7o8G-miR-1 alleviated the hypertrophy, suggesting that oxidative modifications of miR-1 induced by oxidative stress could serve as potential biomarkers and therapeutic targets.^286^ Moreover, oxidative modifications at different positions on miRNA molecules can lead to varied effects on disease progression. For instance, a study on hepatocellular carcinoma demonstrated that an 8-oxoG modification at the third position of miR-122 promotes tumorigenesis, whereas further oxidation of the second nucleotide to yield 2,3o8G-miR-122 transforms it into a tumor-suppressive miRNA.^287^ These findings underscore the intricate mechanisms of nucleic acid functional regulation by oxidative modifications, providing valuable insights for studying molecular mechanisms in disease progression and developing targeted therapeutic strategies.

## Redox determinants of protein degradation

**Fig. 4:**
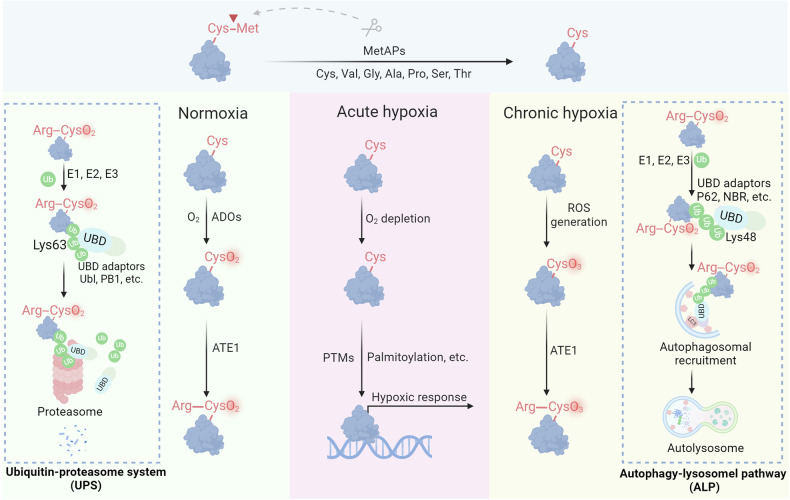
Redox regulation of proteolysis. Under normoxia, Cys2 enzymatically reacts with molecular oxygen (O_2_), followed by arginylation mediated by ATE1. The resulting Arginyl-CysO_2_(H) is recognized by the UBR boxes of N-recognins UBR1 and UBR2, leading to K48-linked ubiquitylation and subsequent proteasomal degradation. During acute hypoxia, oxidation of Nt-Cys2 is either delayed or disrupted, resulting in the stabilization of RGS proteins, which participate in cellular responses to hypoxia. In the context of chronic hypoxia and oxidative stress, Nt-Cys undergoes chemical oxidation by ROS to form CysO_3_(H). Subsequently, ATE1 catalyzes the arginylation of CysO_3_(H), which assembles K63-linked Ub chains on the substrates and degraded by the autophagy-lysosomal pathway

The processes of Alzheimer’s disease and Parkinson’s disease are mainly linked to disrupted systems that break down proteins.^288–290^ When cells produce proteins, they clear improperly folded or redundant proteins to maintain intracellular protein homeostasis.^291,292^ When cells accumulate large amounts of misfolded peptides and oxidatively damaged proteins, protein degradation systems are required to degrade them to maintain cellular homeostasis. Two main pathways for protein degradation exist: the ubiquitin‒proteasome system (UPS) and the autophagy-lysosome pathway (ALP) (Fig. 4).^290,293^

### The ubiquitin-proteasome system

UPS is an essential process for breaking down cell proteins, involving a series of enzymes and approximately 500–1000 proteins.^294,295^ The ATP-dependent proteolytic mechanism operates by the combined effects of three main enzymes: the E1 enzyme activating ubiquitin (Ub), the E2 enzyme for conjugating Ub, and the E3 enzyme for ligating Ub.^296,297^ Collectively, these enzymes facilitate the ubiquitylation of protein substrates, a process critical for targeting proteins for degradation.^298,299^ The human genome encodes more than 600 E3 ligases, underscoring the vast diversity and specificity of substrates targeted by the UPS.^300^ This system targets misfolded or damaged proteins and regulates the levels of numerous short-lived proteins, playing a pivotal role in maintaining cellular function and homeostasis.^301^ The UPS plays a crucial role in multiple cellular functions, such as regulating the cell cycle, transmitting signals, and modulating immune reactions, underscoring its importance in both healthy cell activities and pathological conditions.

The Arg/N-degron pathway, previously known as the Arg/N-end rule pathway, is crucial for controlling protein degradation through the UPS interface and autophagy.^302^ As discovered in 1986, this pathway facilitates the breakdown of proteins and nonprotein entities, such as subcellular organelles and pathogens, through N-degrons.^303^ Specific N-recognin E3 ubiquitin ligases, such as UBR1, UBR2, UBR4/p600, and EDD/UBR5, target N-degrons located at the N-termini of proteins through conserved UBR boxes.^304^ Key N-degrons consist of a positively charged N-terminal Arg, Lys, and His (type 1), as well as bulky hydrophobic Trp, Phe, Tyr, Leu, and Ile (type 2).^305^ The binding of N-recognins to these degrons triggers ubiquitylation and subsequent proteasomal degradation. N-terminal-Arg (Nt-Arg), a key N-degron, is produced by the arginylation of Nt-Asp or Nt-Glu through ATE1-encoded Arg-tRNA transferases or by the deamidation of Nt-Asn and Nt-Gln by specific amidases, as highlighted in the literature.^306^ Additionally, Nt-Cys becomes arginylation-compatible following oxidation, illustrating its role in regulating protein functions through redox reactions and metal ion interactions.

In 2002, the Cys/N-degron pathway was shown to act as an oxygen detector in cardiovascular signaling. This process begins with the oxidation of N-terminal Cys2 and then proceeds with arginylation and proteasomal breakdown.^307^ Important molecules such as RGS4, RGS5, and RGS16, which serve as GTPase-activating proteins for G protein α-subunits, are subject to oxygen-dependent breakdown facilitated by the Met-Cys motif. Typically, Nt-Met is removed by Met aminopeptidases, which expose Cys2 for oxidation and subsequent arginylation.^308^ The resulting Arg-CysO_2_(H) acts as an N-degron, which is identified by UBR1 and UBR2 for ubiquitylation and subsequent degradation. Under hypoxic conditions, the oxidation of Nt-Cys2 is impaired, stabilizing RGS proteins that then suppress G protein signaling, which is critical for cardiovascular function.^309^ This pathway allows cells to adapt to varying oxygen levels by regulating essential proteins, potentially including the proinflammatory cytokine interleukin-32 (IL-32), which is also degraded via this mechanism.^310^ The full scope of proteins affected by this pathway, estimated at approximately 300 in the mammalian genome, remains to be explored.

Transcription factors such as group VII ethylene response factors (ERFs), the polycomb repressive complex 2 component VRN2, and LITTLE ZIPPER2 (ZPR2) are examples of substrates containing an Nt-Cys2.^311^ Under typical oxygen conditions, Nt-Cys2 undergoes oxidation catalyzed by either plant cysteine oxidase 1 (PCO1) or PCO2, followed by arginylation by ATE1, resulting in the formation of Nt-Arg-CysO_2_(H) (RC^O2^).^312^ The N-degron is identified by the N-recognin PRT6, resulting in ubiquitination and degradation by the proteasome.^313^ Under hypoxia, these transcription factors are stabilized and activate hypoxic/anoxic responses by binding to hypoxia-responsive elements. This route enables plants to react to low oxygen levels, such as being submerged or exposed to cold temperatures.^314^ In contrast, *Saccharomyces cerevisiae* does not rely on this pathway, suggesting oxygen sensing might not be as critical in lower eukaryotes.^315^

### The autophagy-lysosome pathway

ALP is a critical component of the cellular protein quality control system, acting as a secondary line of defense for degrading misfolded proteins and other harmful cellular debris.^316,317^ Although the UPS is the primary way to eliminate misfolded proteins from different cell parts, such as the cytosol, nucleus, and ER, it may not always efficiently break down all abnormal proteins.^318^ Proteins that escape monitoring by the UPS can clump together, creating harmful substances that are difficult for the proteasome to break down and remove.^319^ When such aggregates form, the ALP takes over, providing an alternative degradation route to handle these more significant, complex structures.^320^ The ALP is involved in various processes, such as microautophagy, chaperone-mediated autophagy, and macroautophagy.^321–323^ Each mechanism differs in how cellular cargo is delivered to the lysosome for breakdown. Macroautophagy, commonly referred to simply as autophagy, is particularly significant. This process includes trapping items such as misfolded protein clumps, impaired cell parts, and even intruding pathogens in autophagosomes, which are double-membrane vesicles.^324^ These autophagosomes subsequently fuse with lysosomes, forming autolysosomes where lysosomal enzymes degrade the sequestered material. Thus, autophagy is an essential protective mechanism that safeguards cells from various intracellular and extracellular threats, thereby maintaining cellular integrity and function.

In 2021, a study revealed that Nt-Cys triggers the degradation of proteins and other autophagic cargoes, including misfolded proteins, through cis- and trans-pathways to maintain quality control.^325^ The Ub chains direct substrates toward autophagic breakdown through the UBA domain of p62, aiding in the elimination of harmful substances during periods of stress.^326^ Research has demonstrated that autophagy promotes longevity by mitigating the deleterious effects of cellular senescence and accumulation of damaged biomolecules, which are hallmarks of neurodegenerative diseases.^327–329^ Enhanced autophagy has been observed to correlate with increased lifespan in several model organisms, including yeast, nematodes, and flies.^330–332^ The integrin/PI3K/Akt/mTOR pathway is influenced by oxidative stress, leading to the activation of autophagy and accelerating the pathologic process.^333,334^ Additionally, hypoxia-induced oxidative stress stimulates autophagy by inhibiting mTOR activity.^335^ However, various studies have shown that oxidative alterations to autophagy regulators exert an inhibitory effect.^336,337^ For example, oxidation of Cys292 and Cys361 on ATG4B hinders its capacity to degrade the microtubule-associated protein 1A/1B light chain 3 (LC3), leading to a disruption in autophagic flux.^338^ Likewise, the oxidation of Cys263 on ATG3 and Cys572 on ATG7 hinders the attachment of phosphatidylethanolamine (PE) to LC3.^339^ This contradictory outcome may be attributed to intracellular negative feedback regulatory mechanisms that maintain the equilibrium of protein homeostasis. The specific molecular mechanisms may require more refined redox assays and more rigorous experimental designs for further exploration.

## Redox signaling in metabolic reprogramming

**Fig. 5:**
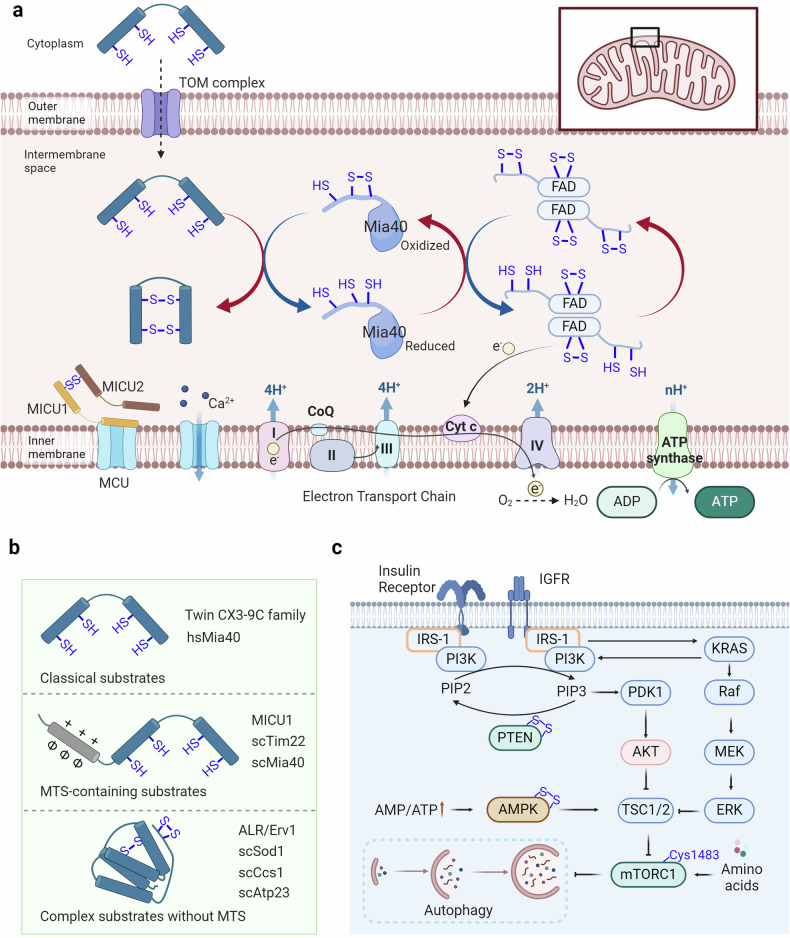
Redox signaling modulates metabolic reprogramming. **a** Proteins enter mitochondria through the intermembrane space via the disulfide relay mechanism. Substrates traverse the mitochondrial outer membrane via the TOM complex, where their free thiols are oxidized by Mia40, facilitating their translocation across the membrane or insertion into the inner membrane. The disulfide bonds of Mia40 are subsequently transferred to FAD, which then transfers its free electrons to cytochrome c. These electrons are then consumed by the electron transport chain to reduce O_2_ and generate H_2_O. **b** Disulfide relay substrates can be categorized into three groups: classical substrates, MTS-containing substrates, and complex substrates without MTS. **c** The insulin receptor and insulin-like growth factor receptor can activate the PI3K-AKT pathway and the RAS-RAF-MEK-ERK pathway, with PTEN regulating PI3K activation. The upregulated ratio of AMP to ATP can activate AMPK, thereby influencing the activation of TSC1/2. Free amino acids can activate mTORC1, thus impacting cell growth and the occurrence of autophagy

Metabolic reprogramming plays a critical role in various diseases, including obesity, fatty liver disease, hyperlipidemia, hypertension, hyperglycemia, gout, cancer, and cardiovascular and cerebrovascular diseases, by adapting cellular energy mechanisms to meet changing physiological needs over time.^340,341^ Recent research, integrating metabolic features from 233 circulating metabolites in 136,016 samples, has revealed the associations between metabolic characteristics and disease onset. Significantly, these metabolic features can characterize the onset of diseases such as diabetes, renal failure, and benign uterine tumors.^342^ As diseases process, there is a shift in the balance between glycolysis and oxidative phosphorylation in cells, which is highly correlated with mitochondrial function.^343–345^ Additionally, pathology-related changes in metabolism are closely linked to the regulation of lifespan and diseases. Metabolic pathways, influenced by nutrient-sensing mechanisms and hormonal changes, dictate cellular responses, influencing both energy production and the cellular maintenance systems.^346,347^ Redox signaling participates in regulating these processes by modulating the activity of critical regulators (Fig. 5).^348,349^ Through its influence on both mitochondrial function and nutrient-sensing pathways, redox signaling helps dictate how effectively a cell can respond to metabolic demands and environmental stress, impacting the pathological process.

### Redox control of mitochondrial dysfunction

Mitochondria are a significant source of ROS, acting as vital organelles for controlling inflammation and triggering cell death.^350,351^ When diseases advance, mitochondrial function deteriorates due to multiple factors, impairing the ability of mitochondria to generate ATP, increasing ROS production, and potentially enhancing mitochondrial membrane permeability, thus promoting inflammation and cell death.^352–354^

Current clinical evidence suggests a beneficial therapeutic effect of L-carnitine on frailty symptoms in older men, possibly achieved through inhibiting fatty acid oxidation in mitochondrial membranes.^355^ Experimental studies in aged mice have shown that inhibiting mitochondrial ATP synthesis using TPP-thiazole can improve metabolic health and delay aging phenotypes.^356^ Metformin has also been demonstrated to exert anti-cancer effects by binding to mitochondrial respiratory chain complexes.^357,358^ However, no direct evidence indicates that inhibiting mitochondrial function can increase human healthspan or lifespan.

Protein production in cells involves the synthesis of polypeptides by cytosolic ribosomes and their subsequent folding into functional structures.^359^ Synthesis and folding can occur simultaneously, as ribosomes enlist different chaperone systems to facilitate the correct folding process within their polypeptide exit tunnels.^360^ Disulfide formation in proteins is reversible, as two cysteine thiols are oxidized into a linked disulfide via thiol-disulfide exchange or oxygen-dependent reactions, often requiring catalysts such as transition metals or flavin adenine dinucleotide (FAD) due to kinetic barriers.^361,362^ Recent findings indicate a high presence of proteins containing structural disulfides in the intermembrane space of mitochondria, leading to a thorough examination of the key elements responsible for oxidizing mitochondrial proteins.^363^ The disulfide relay system within the mitochondria, essential for creating disulfide bonds in proteins, is critical in facilitating the movement of recently produced intermembrane space (IMS) proteins through the mitochondrial outer membrane.^364,365^ Central to this process are Mia40 and Erv1. The localization of Mia40, an IMS protein that is conserved, differs between mammals and plants, where it is soluble, and fungi, where it is membrane-anchored.^366^ Containing a crucial redox-active disulfide within a cysteine-proline-cysteine pattern, this element oxidizes cysteine residues in new polypeptide chains, securing them in a firmly folded condition to block their return through the membrane.^367,368^ This results in direct transport into the IMS. Erv1, a FAD-containing sulfhydryl oxidase family member that includes enzymes in the secretory pathway and viral proteins, reoxidizes Mia40.^369^ Erv1 utilizes two crucial cysteine-x-x-cysteine pairs to transfer electrons from Mia40 to FAD.^367^ In vitro, Erv1 can be reoxidized by oxygen to generate hydrogen peroxide, but in vivo, it primarily transfers electrons to cytochrome c, connecting to the respiratory chain and improving reoxidation efficiency while inhibiting hydrogen peroxide buildup in the IMS.^370,371^ The mitochondrial oxidation machinery also includes Hot13, a small metal-binding protein, further integrating the complex network of mitochondrial protein management.^372^

Mitochondria serve as crucial hubs within the cellular calcium signaling network, where calcium ions participate in mitochondrial and cellular physiological processes intricately linked to cellular energy metabolism and survival.^373–375^ The transport of calcium ions across mitochondria is mediated by the mitochondrial calcium uptake family member protein (MICU). Evidence suggests that various diseases, including cardiovascular and neurological disorders, are closely associated with this process.^376–378^ Recent studies have revealed that MICU3 can form a disulfide bond with MICU1 at the Cys515 site, thereby enhancing calcium uptake by mitochondria.^379^ This suggests that the redox modification of MICU3 may finely regulate mitochondrial calcium influx, consequently modulating the progression of human diseases.

### Redox signaling involved in nutrient-sensing mechanisms

The nutrient-sensing network is integral to how cells perceive and respond to their nutritional environment, directly impacting pathologic processes.^380–382^ It includes receptors that sense intracellular cascades, such as AMPK, mTOR, RAS-MEK-ERK, and PI3K-Akt.^383,384^

AMPK is a crucial protein in cellular sensing of energy metabolism. When the AMP or ADP to ATP ratio significantly increases within the cell, AMPK is activated to maintain energy homeostasis in the organism.^385,386^ In response to energy stress, AMPK restores ATP levels by inhibiting biosynthetic pathways that consume ATP, such as glycogen and lipid synthesis, while activating catabolic pathways that regenerate ATP through macromolecule degradation, which is highly related to human diseases.^387–389^ Oxidation-reduction modifications also participate in the regulation of AMPK activation, where Cys130 and Cys174 of AMPK can form disulfide bonds through oxidation, thereby impeding the binding of AMPK kinase to AMPK and subsequent AMPK activation.^390^ Therefore, the reduction of AMPK oxidation-reduction sites is also one of the essential conditions for AMPK activation.^391^

Furthermore, mTOR, particularly mTORC1, is crucial for regulating cellular metabolism and growth by detecting and combining different nutritional and environmental signals.^392–394^ mTORC1 is sensitive to nutrients, such as glucose and amino acids, and is responsive to cellular stressors such as hypoxia and energy depletion, positioning it as a central regulator of cellular function.^395,396^ Research has shown a strong connection between the function of nutrient detection systems and longevity in both humans and different animal species.^397,398^ Research has shown that under oxidative stress conditions, mTOR can undergo oxidation at Cys1483, forming intermolecular disulfide bonds, thereby inhibiting cardiomyocyte survival and mitochondrial function.^399^

The extracellular signal-regulated kinase (ERK) pathway is a fundamental signaling cascade that transduces external signals into intracellular responses, guiding cellular processes such as proliferation, differentiation, and survival.^400,401^ Oxidative stress can cause the oxidation of tyrosine-protein kinase Fyn at Cys488, leading to the initiation of the Ras/Raf/MEK/ERK cascade.^402^ Current research has focused on understanding how ERK1/2 controls the regulation of more than 300 substrates during oxidative stress.^403^ Recent studies have indicated that oxidative changes at vulnerable cysteine sites, such as Cys38, Cys159, Cys161, Cys183, and Cys214, are involved in the precise control of the ERK1/2 pathway.^404^ R-SO2/3 modification of Cys38 and Cys214 enhances the interaction between MEK and ERK1/2, increasing the phosphorylation of ERK1/2 by MEK.^405^ Moreover, the alteration of Cys159 through R-SOH enhances the formation of ERK2 crystals when ATP is present.^404^ On the other hand, altering Cys183 through R-SNO reduces the phosphorylation of ERK1/2 at both T183 and Y185, leading to apoptosis when cells are treated with sodium nitroprusside.^406^ Previous research has highlighted the varied effects of redox alteration on the ERK pathway. The PI3K/Akt pathway, another nutrient-sensing-related pathway, is upregulated due to the oxidation of PTEN at Cys124 and Cys71.^407^ The PI3K/Akt pathway is crucial in sensing nutrients and regulating cellular metabolism, controlling cellular functions such as growth, survival, and metabolism.^408,409^ This pathway is pivotal in translating extracellular nutrient signals into appropriate intracellular responses, ensuring cellular homeostasis and adaptation to environmental changes.^408^

## Redox regulation of disease microenvironment

**Fig. 6:**
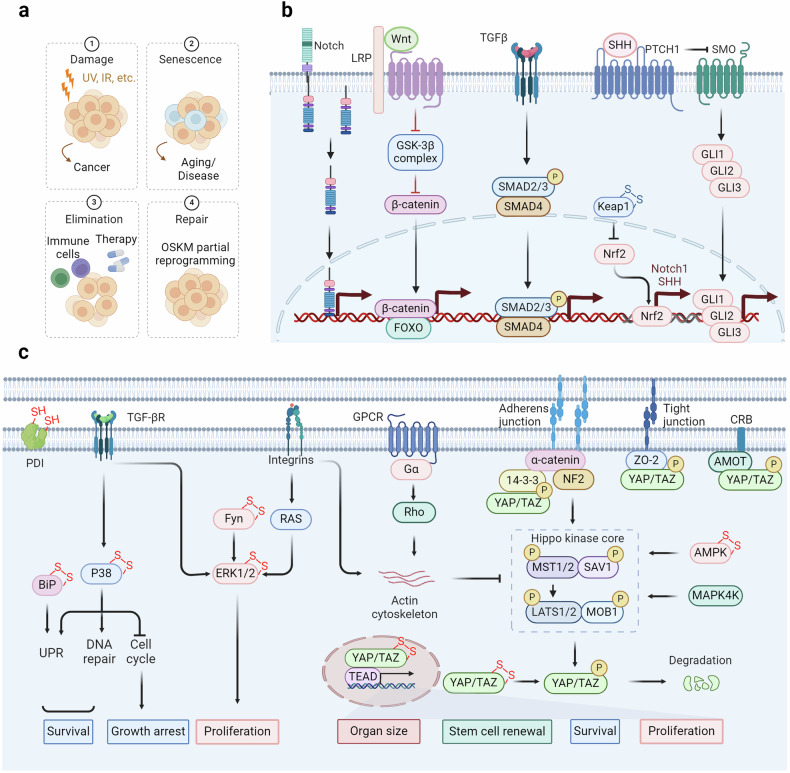
Stem cell exhaustion and intercellular communication regulated by redox signaling. **a** Cellular senescence typically facilitates tissue repair following injury and shields the organism from oncogenic harm. This process occurs through four sequential steps: damage, senescence, elimination, and repair. Failure in each of these steps renders the organism susceptible to developing diseases. **b** The stemness of cells is regulated by multiple pathways. Among them, the redox modification of Keap1 regulates the stability of NRF2, thereby modulating the expression of its downstream effectors Notch1 and Shh. Notch1 and Shh serve as ligands of the Notch and Hedgehog pathways, respectively, and their activation is crucial for maintaining cellular stemness. In the Wnt pathway, FOXO is subjected to redox regulation, affecting the transcription of β-catenin. The TGF-β pathway is also implicated in cellular stemness. **c** The redox signaling pathways regulate extracellular signaling pathways. TGF-β and integrins respectively modulate the activation of p38 and ERK, thereby regulating the proliferation and survival of tumor cells. The Hippo signaling pathway is regulated by adherens junctions, tight junctions, and CRB, which prevent nuclear translocation by binding to phosphorylated YAP. GPCRs and integrins can regulate the actin cytoskeleton, and inhibit the process of the Hippo kinase core, thereby reducing YAP phosphorylation, promoting its nuclear translocation, and regulating organ size, cell survival, and proliferation

The transition from cellular to organismal pathogenesis often necessitates consideration of numerous extracellular factors.^410,411^ Following the emergence of pathologic cells within an organism, their clearance is typically mediated by the immune system, with tissue repair conducted by stem-like cells.^412–414^ In cases where the immune system fails to eliminate senescent cells, chronic inflammation can contribute to a range of age-related diseases, including but not limited to cancer,^415,416^ through the persistent presence of senescence-associated secretory phenotype (SASP).^417,418^ Additionally, intercellular communication and changes in the extracellular matrix significantly influence or activate relevant signaling pathways.^419,420^ The chronic inflammatory microenvironment is one of the critical inducers of diseases, where the activation of inflammatory signaling pathways can provoke the formation of an inflammatory milieu, accelerating the progression of diseases.^421,422^ Within these biological processes, redox signaling regulation can activate relevant pathways by modulating essential redox sensors, thereby promoting the onset and progression of diseases (Fig. 6).

### Redox signaling regulates cellular senescence and stem cell exhaustion

Cellular senescence, triggered by sudden or prolonged damage, leads to the buildup of aged cells in different body parts. It increases significantly (2–20 times) from young (<35 years) to old (>65 years) individuals.^423–425^ Fibroblasts, endothelial cells, and immune cells are mainly impacted by this process, but all types of cells can undergo senescence as they age, which is frequently caused by the shortening of telomeres.^426,427^ The causes of the initial senescence process include oncogenic signaling, genotoxic harm, extremely short telomeres, malfunctioning mitochondria, infections, oxidative stress, nutrient imbalances, and physical stress.^428^ Furthermore, secondary senescence can occur due to inflammatory and fibrotic factors such as CCL2, IL-1b, IL-6, IL-8, and TGF-β.^429,430^ Primary and secondary senescence show biological differences, but their precise molecular differences are unclear.^431^ Cellular senescence is associated with a range of non-proliferative conditions, including lung fibrosis, kidney diseases, liver steatosis, metabolic syndrome related to obesity, type I and II diabetes, atherosclerosis, and neurodegenerative diseases such as Alzheimer’s and Parkinson’s disease.^432–434^ Senescence is believed to play a significant role in these conditions due to SASP.^435,436^

Researchers are particularly interested in the biological function of cellular senescence, despite its connection to other diseases.^437–439^ While primarily acting as a tumor suppressor, cellular senescence also plays a critical role in tissue repair, promoting localized fibrosis and immune cell recruitment to clear damaged and senescent cells.^440–442^ This process can be viewed as a two-phase mechanism: initial senescence induction followed by immune-mediated clearance.^443–445^ Senescence is a self-limiting response that, ideally, leads to beneficial outcomes.^446^ However, pathological effects emerge when immune clearance fails, leading to senescent cell accumulation and SASP-driven fibrosis in the tissue microenvironment.^447,448^ In this context, the redox signaling pathways play a crucial regulatory role, and their specific molecular mechanisms await further exploration.

Aging diminishes tissue renewal and impairs repair across organs, each employing unique strategies.^449–451^ Conversely, the skin epidermis features multiple stem cell niches, especially around hair follicles, facilitating high renewal rates and progeny generation.^452^ Upon injury, cells in these niches can adopt stem cell characteristics and overcome established boundaries.^453^ Conversely, the liver, lung, and pancreas usually have low regeneration rates under normal circumstances. Nevertheless, they can gain stem-like properties, including proliferation and multipotency, in response to injury.^454^ Tissue repair significantly depends on injury-induced cellular dedifferentiation and plasticity.^455^ Damage to the intestine, brain, and lungs causes non-stem cells to revert to an earlier state, activating genetic programs related to embryonic development and stem cell characteristics to facilitate repair flexibility.^456,457^ Recent multi-omics studies revealed that glutathione metabolism is a key determinant of bimodal patterns in stem cell exhaustion, indicating the essential role of redox state in stemness regulation.^458^ Maintaining a stem-like phenotype necessitates the activation of diverse signaling cascades, including the Wnt, Notch, and Hedgehog pathways.^459–462^ ROS help transport the FOXO transcription factor into the nucleus through phosphorylation by JNK or MST1.^463^ Subsequently, FOXO may interact with β-catenin, the pivotal regulator within the Wnt pathway, culminating in the transcriptional upregulation of genes associated with cell cycle arrest, antioxidative defense mechanisms, and DNA repair processes.^464^ Furthermore, the generation of ROS can activate NRF2, leading to the transcriptional activation of Notch1 and Sonic Hedgehog, which in turn stimulates the Notch and Hedgehog signaling pathways.^465,466^ This activation is predominantly mediated through the disruption of the Kelch-like ECH-associated protein 1 (KEAP1) - NRF2 interaction (oxidation of KEAP1 Cys151, Cys273, and Cys288),^29^ facilitating the translocation of NRF2 into the nucleus.

### Role of extracellular matrix in redox regulation

**Fig. 7:**
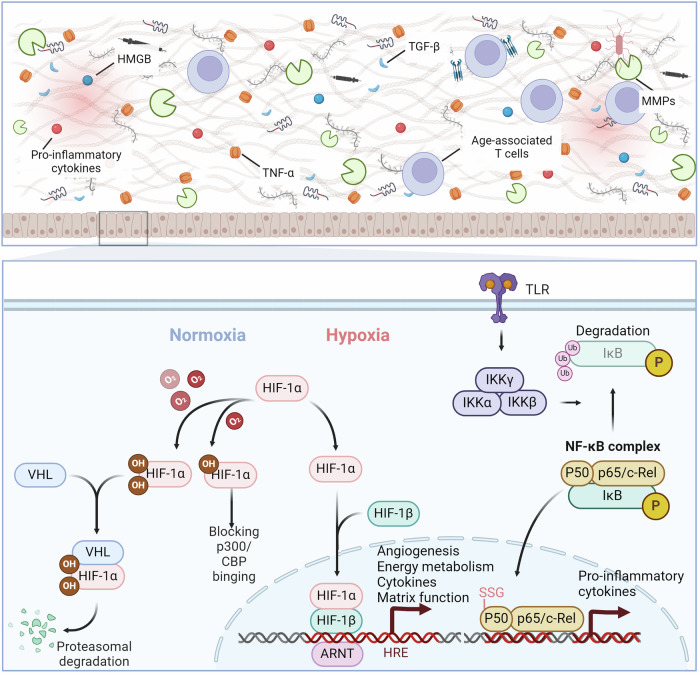
Redox signaling regulates the disease-related microenvironment. Under normoxic conditions, HIF-1α undergoes oxidative modifications, leading to its degradation through binding to VHL or sequestration in the cytoplasm via binding with p300. In hypoxic conditions, oxidative modifications decrease, allowing for nuclear translocation of HIF-1α and the transcriptional activation of genes related to angiogenesis, energy metabolism, cytokines, and matrix function. Activation of Toll-like receptors leads to the phosphorylation and degradation of IκB, releasing NF-κB complexes for nuclear transcription of genes associated with inflammatory factors. The p50 subunit can be modified by glutathionylation, hindering its binding to DNA. Inflammatory cytokines, TGF-β, HMGB, and other factors regulate the formation of the inflammatory microenvironment

The extracellular matrix (ECM) is a ubiquitous three-dimensional, non-cellular structure found across various tissues, serving not only as physical support but also as a dynamic network.^467^ This network regulates a variety of functions including proliferation, migration, and differentiation through interactions with cells.^468,469^ Enhancing our understanding of how the ECM influences organ structure and functionality, as well as how ECM remodeling impacts disease progression, could aid in developing new therapeutic approaches.^470,471^ The ECM is particularly critical in the development of specific organs like the intestines, lungs, and mammary glands, and its remodeling also impacts their normal morphogenesis (Fig. 7).^472–474^ Integrins are crucial transmembrane receptors that mediate the interaction between cells and the ECM, playing a vital role in cellular signaling and structural integrity.^475,476^ The relationship between integrins and the ECM is highly dynamic and regulated by several mechanisms, including conformational changes of integrins, which can switch between inactive and active states.^477,478^ Integrins bind to ECM components such as collagen and fibronectin in the active state, initiating intracellular signaling cascades.^479,480^ This activation often involves focal adhesion kinase (FAK), which is a critical mediator in transducing signals from integrins to promote cell cycle progression and proliferation through pathways like Ras-ERK/MAPK.^481,482^ Under oxidative stress conditions, integrins can form disulfide bonds (e.g., Cys560-Cys583 and Cys523-Cys544 in the β3 subunit) that lock them in an inactive, bent conformation, preventing them from engaging with the ECM.^483^ Moreover, the ECM itself, which includes components like collagen, laminin, and glycoproteins, is susceptible to oxidation.^484–486^ Oxidative modifications can lead to structural and functional changes in these proteins, further impacting the integrin-ECM interaction and influencing cellular behavior.

The increase in TGF-β and other growth factors, along with the activation of the transcription factors TAZ and YAP, contributes to the promotion of the expression of profibrotic genes, such as transglutaminase-2 and LOX.^487,488^ Increased ECM rigidity worsens the functions of senescent cells by boosting the secretion of matrix metalloproteases, intensifying ECM damage, and triggering pathways that promote senescence, fibrosis, and inflammation.^489,490^ Increased ECM stiffness enhances WNT signaling, leading to fibroblast activation and profibrotic gene expression.^491,492^ The pathway crosses paths with NOTCH, RAS, TGF-β/SMAD, and hedgehog/GLI, demonstrating the intricate interaction involved in the development of fibrosis.^493,494^ Additionally, matrix stiffness-induced mechanical changes impair oligodendrocyte progenitor cell function via the PIEZO1 ion channel.^495^ Studies have shown that the activation of TGF-β by ROS can effectively initiate the restructuring of the ECM. The oxidation of Met253 on the latency-associated peptide β (LAP-β) disrupts its interaction with TGF-β1, leading to the activation of latent TGF-β1 ligands.^496,497^

The Hippo signaling pathway, which was initially identified through studies in Drosophila, is a critical regulator of organ size and tissue homeostasis in mammals.^498,499^ It regulates various cellular activities, including growth, cell death, and the ability of stem cells to reproduce themselves.^500–502^ Recent research has expanded our understanding of the Hippo pathway, highlighting its role in regulating the ECM.^503,504^ YAP acts as a redox detector, identified by two regions rich in cysteine, the cysteine-rich domain at the end of the molecule (C-CRD) with redox-responsive Cys598, Cys620, and Cys629 and the cysteine-rich domain at the beginning of the molecule (N-CRD) with Cys303, Cys310, and Cys315.^505^ YAP can assume various oxidized states depending on the intensity and duration of oxidative stress. YAP quickly creates an intradomain disulfide bond between Cys310 and Cys315 (disulfide bond 1) within 10 s to 5 min under 0.5 mM H_2_O_2_ exposure.^506^ Subsequently, around the 5-min mark, YAP forms an interdomain disulfide bond (disulfide bond 2) and converts disulfide bond 1 into another interdomain disulfide bond 3. After prolonged treatment, YAP ultimately forms a fourth disulfide bond.^507^

The p38 MAPK pathway is an important controller of cellular functions and plays a key role in regulating the extracellular matrix.^508,509^ p38 MAPK is involved in fibrotic processes by promoting the differentiation of fibroblasts into myofibroblasts, which are central to wound healing and fibrosis.^510^ This differentiation is typically associated with increased synthesis of ECM components, which contributes to the stiffening and scarring of tissues. p38 MAPK activity leads to enhanced production of type I collagen by myofibroblasts, a key event in the development of fibrosis in organs such as the liver and lungs.^511,512^ Recent research has shown that the activity of p38 is influenced not only by the interplay of upstream regulatory kinases and phosphatases but also by the incorporation of redox modifications on p38α.^513^ These modifications allow dynamic modulation of the interactions between p38 and its upstream activator, MKK3.^514^ Specifically, oxidative modifications at several cysteine residues within p38, including Cys39, Cys119, Cys162, and Cys211, occur following exposure to oxidative stressors such as H_2_O_2_ or the ROS inducer prostaglandin J2. Notably, Cys119 and Cys162 have been identified as the principal sites for oxidation and significantly influence the regulatory dynamics of MKK3.^515^ The p38 signaling pathway is influenced by oxidative regulation, emphasizing the important connection between the cellular redox balance and related signaling pathways. Persistent oxidative stress and ROS play crucial roles in the onset of diseases.

Protein disulfide isomerase (PDI) catalyzes disulfide bond rearrangement and has been reported to exist on the cell surface and in the ECM, exhibiting dysregulated expression under pathological conditions.^516–518^ The CGHC motif in its molecular structure allows it to reduce disulfide bonds in other proteins containing them.^519^ Studies have shown that PDI can modulate the oxidation-reduction modification of integrin αvβ3, thereby regulating its protein conformational changes.^520,521^ Another adhesion receptor, GPIbα, can also undergo reduction of its intramolecular disulfide bonds Cys4-Cys17 and Cys209-Cys248 by PDI.^522^ PDI can also directly regulate ECM proteins; for example, it can reduce the disulfide bonds Cys137-Cys161 and Cys274-Cys453 in vitronectin, thereby promoting its interaction with integrin β3.^520,523^

### Chronic inflammation and redox imbalance

The intricate interplay between redox signaling pathways and chronic inflammation is pivotal to understanding the pathogenesis of chronic diseases.^524,525^ This state of chronic, low-grade inflammation is strongly associated with various diseases and pathologies, including arteriosclerosis, neuroinflammation, osteoarthritis, and intervertebral disc degeneration.^526,527^ Oxidative damage caused by inflammation can induce inflammatory reactions through the activation of nuclear factor kappa-light-chain-enhancer of activated B cells (NF-κB), a crucial transcription factor that controls the production of inflammatory cytokines such as TNF-α and IL-6.^528,529^ Research indicates that the Cys62 residue of the p50 subunit of NF-κB can undergo glutathionylation, thereby impeding its binding to DNA and subsequent transcription.^530,531^ Moreover, increased levels of inflammatory cytokines and biomarkers, including CRP, along with elevated IL-6 in the bloodstream, can be used to predict overall mortality in older populations.^532,533^ Furthermore, oxidation-reduction modifications can directly facilitate the binding of IL-2 to its receptor by forming disulfide bonds at the Cys183-Cys232 site, thereby promoting subsequent T-cell proliferation.^534^ High mobility group box 1 (HMGB1) is a nuclear protein released during tissue damage, and intriguingly, its biological functions are regulated by oxidation-reduction modifications.^535–537^ When its Cys106 site is oxidized, it can interact with TLR4 to promote inflammatory responses.^538^ Conversely, when HMGB1 is fully reduced, it can facilitate tissue repair through CXCR4, suggesting the significant role of oxidation-reduction modifications in these processes.^538^ Increased inflammation is accompanied by decreased immune system effectiveness, which can be observed by analyzing myeloid and lymphoid cells in the blood of humans and mice.^539,540^ Age-associated T cells are exhausted memory cells, can exacerbate inflammation through granzyme K.^541,542^ Alterations in T-cell behavior result in heightened proinflammatory TH1 and TH17 responses, reduced immune monitoring affecting the elimination of infected or cancerous cells, diminished self-tolerance contributing to autoimmune conditions, and weakened natural defenses, ultimately fostering widespread inflammation.^543–545^

Redox signaling pathways are central to the regulation of chronic inflammation.^546,547^ HIF-1α is a critical transcription factor that responds to changes in cellular oxygen levels.^548^ Under conditions of normal oxygen levels (normoxia), the activity of HIF-1α is suppressed via redox-dependent modifications. Specifically, prolyl-4-hydroxylases (PHDs) catalyze the oxidation of proline residues at positions 402 and 564 on HIF-1α.^549,550^ This oxidative modification facilitates the recruitment of the von Hippel-Lindau protein (pVHL), which targets the oxidized HIF-1α for polyubiquitination and subsequent proteasomal degradation.^551,552^ Furthermore, the Factor Inhibiting HIF-1 (FIH-1) disrupts the association between HIF-1α and the p300/CBP complex.^553^ This interference occurs through the hydroxylation of the asparagine residue at position 803 on HIF-1α.^554^ In the context of the inflammatory microenvironment, HIF-1α has been shown to regulate the expression of inflammatory cytokines. It enhances the transcription of genes such as IL-6 and TNF-α, which are pivotal in the inflammatory response. However, HIF-1α also upregulates the production of anti-inflammatory cytokines like IL-10, creating a complex regulatory network that can either exacerbate or alleviate inflammation depending on the context. HIF-2α possesses a structure similar to HIF-1α, and its protein stability is also regulated by redox modifications and targeted for degradation by VHL.^555^ However, an evolutionarily conserved amino acid substitution at the binding site for VHL in HIF-2α results in a 2-3-fold decrease in its binding affinity compared to HIF-1α.^556^ Consequently, researchers believe that HIF-1α may serve as a mechanism for cells to rapidly respond to acute hypoxia, whereas HIF-2α may be more relevant in chronic hypoxic responses.^557^ Recent studies have found that the expression of HIF-2α may facilitate the maintenance of a pseudohypoxic state in cells, thereby promoting the onset and progression of diseases, including renal clear cell carcinoma,^558^ pancreatic cancer,^559^ and breast cancer.^560^

The balance between oxidative and antioxidative mechanisms determines the extent of inflammation, influencing the progression of diseases.^561^ Enhancing antioxidant defenses and modulating redox signaling may offer therapeutic potential to mitigate chronic inflammation and improve health outcomes in elderly people.^562,563^ Further research into specific redox mechanisms and their interactions with cellular senescence and immune regulation will be essential for developing targeted interventions against inflammation and its related pathologies.^564,565^

## Clinical strategies targeting redox signaling in disease treatment

Oxidative stress plays a significant role in the onset and progression of various diseases; thus, antioxidant therapy has shown efficacy in the treatment of specific conditions. In 1956, oxidative stress was closely linked to the aging process, and lifespan extension in mice was demonstrated by feeding them with radical scavengers. By 1980, radicals were proven to be a major cause of ischemic reperfusion injury. Consequently, antioxidant therapy has been effective in treating these conditions. However, further research has revealed that the development of some diseases is intricately regulated by redox signaling pathways (Table 1), suggesting that simplistic antioxidant therapy might exacerbate these conditions (Table 2). Redox signaling participates in various aspects of the pathogenesis, suggesting new strategies for treating human diseases.^566–569^ As mentioned above, numerous redox sensors receive redox signals, and their function is altered by specific modifications. Due to their essential function, redox sensors can be developed as therapeutic targets for the clinical treatment of human diseases.^570–572^ However, directly targeting redox sensors for oxidative modification could be challenging in the long term.^573^ With technological advancements, precision modulation of redox modifications is beginning to show promise, building on the foundation of broad-spectrum antioxidant treatment.

**Table 1:** Representative redox sensors in the regulation of human diseases

Protein	Site	Modification	Disease	Function	Ref.
ATM	Cys2991	-S-S-	Ataxia-telangiectasia disease	DNA repair	^93^
RAD51	Cys319	-S-S-	Triple-negative breast cancer	DNA repair (HR)	^101^
XRCC3	Cys86, Cys328	-S-S-	Cancer	DNA repair (HR)	^108^
KU80	Cys493, Cys638	-S-S-	Cancer	DNA repair (NHEJ)	^126^
AAG	Cys167	R-SNO	Cancer	DNA repair (BER)	^144^
APE1	Cys65	-S-S-	Glioma	DNA repair (BER) biological function	^168^
APE1	Cys93, Cys310	R-SNO	Liver cancer	Subcellular localization	^169^
APE1	Cys99	R-SSG	Cervical cancer	DNA binding and endonuclease activity	^170^
XRCC1	Cys12, Cys20	-S-S-	N.A.	Increasing the binding ability to polβ	^176^
MGMT	Cys145	R-GSNO	Glioma	Direct reversal pathway	^187^
MAT	Cys121	-S-S-	Liver diseases	Biosynthesis of SAM	^260^
DnaJb5	Cys274, Cys276	-S-S-	Cardiac hypertrophy	Histone acetylation	^269^
HDAC4	Cys667, Cys669	-S-S-	Cardiac hypertrophy	Histone acetylation	^269^
HDAC8	Cys102, Cys153	-S-S-	Neuroblastoma	Histone acetylation	^272^
RGS proteins	Cys2	Arg-CysO_2_(H)	Osteosarcoma, Colon cancer	Degradation by UPS	^326^
ATG4B	Cys292, Cys361	-S-S-	Cancer	Attenuating autophagic flux	^338^
ATG3	Cys263	-S-S-	Aging	Attenuating autophagic flux	^339^
ATG7	Cys572	-S-S-	Aging	Attenuating autophagic flux	^339^
MICU1	Cys515	-S-S-	Heart failure	Enhancing calcium uptake by mitochondria	^379^
AMPK	Cys130, Cys174	-S-S-	Myocardial ischemia	Inhibiting the activation of AMPK	^391^
mTOR	Cys1483	-S-S-	Myocardial ischemia	Inhibiting the activation of mTOR	^399^
Fyn	Cys488	N.A.	Skin carcinogenesis	Activating Ras/Raf/MEK cascade	^402^
ERK1/2	Cys38, Cys214	R-SO_2/3_	Lung cancer	Promoting interaction between MEK and ERK1/2	^405^
ERK1/2	Cys159	R-SOH	Melanoma	Promoting crystal packing of ERK2 in the presence of ATP	^404^
ERK1/2	Cys183	R-SNO	Breast cancer, Glioma	Decreasing the dual-phosphorylation of ERK1/2 on T183 and Y185	^406^
PTEN	Cys124, Cys71	-S-S-	Cancer	Activation of PI3K/Akt pathway	^407^
LAP-β	Met253	N.A.	Breast cancer	Activating TGF-β1	^497^
YAP	Cys303, Cys310, Cys315, Cys598, Cys620, Cys629	-S-S-	N.A.	Forming 1 to 4 disulfide bonds and activating YAP	^505^
p38 MAPK	Cys119, Cys162	-S-S-	Myocardial ischemia	Promoting the interaction between MKK3	^515^
HIF-1α	Pro402, Pro564	R-OH	Cancer	Recruiting VHL for ubiquitin-dependent degradation	^549^
HIF-1α	Asn803	R-OH	Cancer	Blocking p300/CBP binding	^554^
Integrin β3	Cys560-Cys583, Cys523-Cys544	-S-S-	Thrombosis	Inactivation of integrins	^483^
Vitronectin	Cys137-Cys161, Cys274-Cys453	-S-S-	Thrombosis	Promoting its interaction with integrin β3	^520^
P50	Cys62	-SSG	N.A.	Impeding its binding to DNA	^530^
IL-2R	Cys183-Cys232	-S-S-	N.A.	Facilitate the binding of IL-2 to its receptor	^534^
KEAP1	Cys151, Cys273, Cys288	N.A.	Acute inflammatory responses	Decreasing ubiquitin E3 ligase activity	^29^

**Table 2:** Representative clinical trials treating diseases from a redox perspective

Drug	Indication	Targeted mechanism	Official title	Phase	NCT number
Curcumin and Ginkgo extract	Alzheimer’s Disease	NRF2 activator	A Pilot Study of Curcumin and Ginkgo for Treating Alzheimer’s Disease	Phase II	NCT00164749
Glutathione	Alzheimer’s Disease	Buffering ROS	Glutathione, Brain Metabolism and Inflammation in Alzheimer’s Disease	Early Phase I	NCT04740580
Edaravone	Alzheimer’s Disease	NRF2 activator	Alzheimer Study Using oRal Edaravone	Phase II	NCT05323812
Flos Gossypii Flavonoids	Alzheimer’s Disease	NRF2 activator	Efficacy and Safety of Flos Gossypii Flavonoids Tablet in the Treatment of Alzheimer’s Disease	Phase II	NCT05269173
Lipoic acid and fish oil	Alzheimer’s Disease	NRF2 activator	Fish Oil and Alpha Lipoic Acid in Treating Alzheimer’s Disease	Phase II	NCT00090402
Lipoic Acid and Omega-3 Fatty Acids	Alzheimer’s Disease	NRF2 activator	Lipoic Acid and Omega-3 Fatty Acids in Alzheimer’s Disease	Phase II	NCT01058941
N-acetylcysteine	Parkinson’s Disease	Buffering ROS	Repeated-Dose Oral N-acetylcysteine for the Treatment of Parkinson’s Disease	Phase II	NCT02212678
N-acetylcysteine	Gaucher’s Disease and Parkinson’s Disease	Buffering ROS	Intravenous N-acetylcysteine for the Treatment of Gaucher’s Disease and Parkinson’s Disease	Phase I	NCT01427517
Green Tea Polyphenol	Parkinson’s Disease	NRF2 activator	Efficacy and Safety of Green Tea Polyphenol in De Novo Parkinson’s Disease Patients	Phase II	NCT00461942
Glutathione	Parkinson’s Disease	Buffering ROS	Glutathione in the Treatment of Parkinson’s Disease	Phase II	NCT01177319
Coenzyme Q10	Parkinson’s Disease	NRF2 activator	Parkinson’s Disease Treatment with Coenzyme Q10	Phase II	NCT00004731
N-acetylcysteine	Head and Neck Neoplasms	Buffering ROS	Evaluation Of the Use Of N-Acetylcysteine Attenuating Cisplatin-Induced Toxicities By Oxidative Stress In Head And Neck Cancer Patients	Phase IV	NCT02241876
Flavonoids	Colorectal Cancer	NRF2 activator	Dietary Bioflavonoid Supplementation for the Prevention of Neoplasia Recurrence	Phase II	NCT00609310
Sulforaphane	Bladder cancer	NRF2 activator	Randomized, Phase II Clinical Trial of Sulforaphane in Bladder Cancer Chemoprevention	Phase II	NCT03517995
Curcumin	Prostate Cancer	NRF2 activator	Trial of Curcumin to Prevent Progression of Low-risk Prostate Cancer Under Active Surveillance	Phase III	NCT03769766
GC4419	Radiation-Induced Oral Mucositis	SOD mimic	A Study of the Effects of GC4419 on Radiation-Induced Oral Mucositis in Patients with Head/Neck Cancer	Phase II	NCT02508389
Ebselen	Meniere’s Disease	GPx mimic	SPI-1005 for the Treatment of Meniere’s Disease (STOPMD-3)	Phase III	NCT04677972
ALT-2074	Coronary Artery Disease	GPx mimic	Evaluation of ALT-2074 in Subjects with Type 2 Diabetes, Haptoglobin Type 2-2 Genotype and Coronary Artery Disease	Phase II	NCT00491543
Quercetin	COPD	NRF2 activator	Beneficial Effects of Quercetin in COPD	Phase II	NCT06003270
Erdosteine	COPD	Buffering ROS	The Efficacy and Safety of Erdosteine in COPD	Phase III	NCT01032304
Sulforaphane	COPD	NRF2 activator	Broccoli Sprout Extracts Trial to See if NRF2 is Enhanced by Sulforaphane Treatment in Patients with COPD (BEST)	Phase II	NCT01335971
Fisetin	Peripheral Arterial Disease	NRF2 activator	Fisetin to Reduce Senescence and Mobility Impairment in PAD	Phase II	NCT06399809
Curcumin	Type 2 Diabetes Mellitus	NRF2 activator	Curcumin for Type 2 Diabetic Patients	Phase IV	NCT01052597
Ginkgo Biloba Extract	Type 2 Diabetes Mellitus	NRF2 activator	Ginkgo Biloba Extract and the Insulin Resistance Syndrome	Phase II	NCT00032474
N-Acetylcysteine and Arginine	Type 2 Diabetes Mellitus	Buffering ROS	N-Acetylcysteine and Arginine Administration in Diabetic Patients	Phase IV	NCT00569465
Melatonin	Type 2 Diabetes Mellitus	NRF2 activator	Melatonin’s Effects on the Treatment of Diabetes Mellitus	Early Phase I	NCT02691897
Fenofibrate and Coenzyme Q10	Type 2 Diabetes Mellitus	NRF2 activator	A Randomised, Double-Blind, Placebo-Controlled Study Assessing the Effect of Fenofibrate, Coenzyme Q10 and Their co-Administration on Ventricular Diastolic Function in Patients With Type 2 Diabetes	Phase II	NCT00703482
Curcumin	NAFLD	NRF2 activator	Curcumin for Pediatric Non-alcoholic Fatty Liver Disease	Phase II	NCT04109742
N-Acetylcysteine	NAFLD	Buffering ROS	N-acetylCysteine and Patients With Non-alcoholic Fatty Liver Disease	Phase III	NCT05589584
Lipoic acid	NAFLD	NRF2 activator	Effect of Alpha Lipoic Acid on Non-alcoholic Fatty Liver Diseases	Phase IV	NCT04475276
Coenzyme Q10	NAFLD	NRF2 activator	The Effect of Coenzyme Q10 on Endothelial, Vascular and Myocardial Function	Phase II	NCT05941910
Glucocorticoid and sinomenine	Knee Osteoarthritis	NRF2 activator	Sinomenine Versus Glucocorticoid for Knee OA	Phase III	NCT05764304
Astaxanthin	Knee Osteoarthritis	NRF2 activator	Effect of Astaxanthin in Moderate to Severe Knee Osteoarthritis	Phase II	NCT05437601
Grape Seed Extract	Heart Failure	NRF2 activator	Physiological Effects of Grape Seed Extract in Diastolic Heart Failure	Phase I	NCT01185067
Lipoic Acid	Heart Failure	NRF2 activator	Clinical Study of Lipoic Acid on Ischemic Heart Failure	Phase IV	NCT03491969
N-Acetylcysteine	Heart Failure with Chronic Renal Failure	Buffering ROS	N-Acetylcysteine in Heart Failure with Coexistent Chronic Renal Failure	Phase III	NCT00532688

### Antioxidant therapy

The antioxidant system within organisms primarily relies on the catalytic action of antioxidant enzymes and the sufficient supply of their substrates.^574,575^ Enhancing the biological antioxidant system is a common strategy in antioxidant therapy.^576,577^ Superoxide dismutase (SOD) was the first antioxidant enzyme discovered and remains the only enzyme known to eliminate superoxide anion (O^2•−^) in mammals, playing a crucial role in combating oxidative stress.^578^ Orgotein, the first SOD mimic, exhibits significant anti-inflammatory effects and has shown therapeutic potential in animal studies.^579^ However, due to some side effects, it has not yet been approved for use in humans. Additionally, another SOD mimic, GC4419,^580^ has demonstrated good safety in phase II clinical trials among head and neck cancer patients suffering from severe oral mucositis induced by radiation and chemotherapy (NCT02508389).

Another class of promising antioxidants consists of glutathione peroxidase mimetics, among which ebselen exhibits favorable oral tolerance and bioavailability.^581,582^ It has undergone several clinical trials for various conditions, including Meniere’s disease (Phase III, NCT04677972), bipolar disorder, complete middle cerebral artery occlusion, delayed neurological deficits following aneurysmal subarachnoid hemorrhage, and acute ischemic stroke. ALT-2074 (BXT-51072), a novel analog of ebselen, has completed Phase II clinical trials in the contexts of diabetes and coronary artery disease (NCT00491543).

In addition to the development of enzyme mimetics, research has also focused on the substrates for antioxidant enzymes. NAC can provide GSH as a substrate for antioxidant redox reactions.^583^ NAC has been utilized to treat Parkinson’s disease (NCT02212678),^584^ type 2 diabetes mellitus (NCT00569465),^585^ nonalcoholic fatty liver disease (NAFLD) (NCT05589584),^586^ and heart failure (NCT00532688).^587^ Similarly, glutathione can attenuate oxidative stress, benefiting patients with Alzheimer’s disease (NCT04740580)^588^ and Parkinson’s disease (NCT01177319).^589^ However, its approval for clinical use has not been secured, potentially due to oxidative stress not being the primary causative factor in the diseases studied.

Beyond antioxidant enzyme mimetics, another class of antioxidant therapeutic strategies involves activating the NRF2 antioxidant transcription factor.^590,591^ Extracts from dietary vegetables and fruits can activate the NRF2 signaling pathway and induce the expression of antioxidant enzymes, and some are currently being tested in clinical trials for disease treatment and prevention.^592^ For example, the natural product curcumin for treating Alzheimer’s disease (NCT00164749),^593^ type 2 diabetes mellitus (NCT01052597),^594^ and NAFLD (NCT04109742)^595^ has entered clinical trials. However, the use of NRF2 activators faces certain challenges that limit their approval for therapeutic use in humans. In addition to activating NRF2 and inducing antioxidant enzymes, some NRF2 activators may also affect other signaling pathways, potentially interfering with related biological processes. Moreover, the effects of NRF2 activation and antioxidant induction are not limited to specific cells or organs, which could result in systemic side effects.

Additionally, certain dietary antioxidants can neutralize free radicals under conditions of oxidative stress or antioxidant deficiency, thereby preserving the structural integrity of cellular components.^596^ For example, vitamins such as vitamin C and vitamin E can donate electrons to reduce biologically significant macromolecules that have been oxidatively damaged.^597^ However, due to the limited capacity of small molecule antioxidants to neutralize free radicals effectively, their antioxidant capabilities are significantly inferior to those of antioxidant enzymes. Consequently, their effectiveness in clinical applications often falls short.

Antioxidant therapy has demonstrated promising therapeutic effects in diseases where oxidative stress is a primary pathogenic factor and has entered clinical trials as an adjunct treatment method for various other diseases. However, in complex diseases such as cancer, the efficacy of antioxidant therapy is often controversial and may even promote disease progression. Consequently, current basic research is dedicated to identifying key regulatory proteins and critical redox modification sites involved in the pathogenesis and progression of diseases. This aims to facilitate personalized medicine and develop targeted small molecule drugs that are effective for specific populations.

### Targeting specific redox modification

As summarized above, redox signaling pathways are a regulatory mode of signal transduction that depends on redox modifications, controlling the biological functions of key proteins through reversible or irreversible redox modifications.^598^ Cysteine residues, where redox modifications commonly occur, are ubiquitously present in proteins, particularly enriched in the active sites of various enzymes.^599^ The redox modifications on these residues have potential regulatory implications for enzyme activity and protein structure, which are vital for life processes within organisms.^600^ Recognizing this potential, researchers have investigated cysteines with redox modification capabilities within organisms, conducting screenings under various model conditions and inducements, and identifying numerous candidate sites.^216,601^ However, due to technical limitations and the complexity of proteins in biological systems, research on these sites lacks systematic planning, making it challenging to form conclusive research outcomes. Recent studies have used big data to predict protein conformational changes post-redox modifications, assess cysteine sites with potential ligand-binding capabilities, and subsequently establish the DrugMap database.^602^ Through simulation, chemical probes targeting active cysteine sites in NF-κB and SOX10 have been identified, offering new potential strategies for specifically targeting active cysteine residues. Currently, several small molecule drugs targeting specific cysteine residues of redox sensors have been developed. For instance, Curcumin has been shown to covalently bind to the Cys151 residue of KEAP1, thereby stabilizing NRF2 and enhancing antioxidant effects.^603^ Building on this, researchers have further optimized the structure of curcumin to develop more efficient small molecules that covalently bind to KEAP1.^604^ Furthermore, BI-78D3 targets the Cys159 site on ERK2, thereby inhibiting ERK2 signal activation.^605^ This compound has been validated in mouse models for its potential to treat melanoma. Targeting the active cysteine sites of critical proteins in drug development promises to reduce the toxic side effects associated with off-target effects, while also providing more pronounced inhibitory effects. Validating the safety and biological functions of candidate drugs will aid their progression toward clinical trials and applications.

## Concluding remarks

In this review, we have summarized the mechanisms through which redox signaling pathways regulate human diseases as well as the current clinical potential of therapeutic strategies. However, when confronted with external stimuli, dysregulation often occurs, leading to the onset of diseases. Cells utilize oxidative stress signaling as a quick response mechanism to combat external triggers within the body. With recent advances in our understanding of the oxidative-reductive signaling network, it has been revealed that ROS are not only metabolic byproducts but also critical second messengers within cells that regulate the biological functions of numerous vital proteins. Redox signaling pathways can counteract the effects of external influences on the genome and epigenome by regulating DNA repair and vital epigenetic proteins. Furthermore, the protein degradation regulatory system is finely controlled by redox signals, thereby managing the degradation and recycling of damaged or misfolded proteins within cells. Cellular nutrient-sensing pathways and essential energy metabolism organelles, such as mitochondria, are also regulated by redox signaling pathways, which control cellular nutrient intake and metabolism. At the tissue level, redox signaling pathways also regulate changes in the extracellular matrix and microenvironment, promoting the timely clearance of senescent cells and preventing the onset of human diseases. Therefore, redox regulation is involved in the occurrence and progression of various diseases at multiple levels and is expected to become a key entry point for therapy.

Antioxidant therapy has shown satisfied efficacy in diseases where oxidative stress is a primary pathological factor, including atherosclerosis, radiation-induced lung injury, and paraquat poisoning. It has also been widely applied in diseases caused by complex pathogenic factors, such as Alzheimer’s disease, NAFLD, and cancer. Since the rate at which antioxidant enzymes eliminate oxidative free radicals in biological systems far exceeds that of antioxidant small molecules, most current antioxidant therapies focus on the strategy of regulating antioxidant enzymes. For example, SOD, the only antioxidant enzyme that eliminates superoxide anions in mammals, has analogs that effectively inhibit redox damage to biological macromolecules. After multiple iterations and improvements, several analogs have progressed to clinical trials. Additionally, glutathione peroxidase mimetics have also entered clinical trials as analogs of antioxidant enzymes. Substrates of antioxidant enzymes, such as NAC, have demonstrated good therapeutic effects in the treatment of various diseases. Furthermore, numerous effective small molecules targeting NRF2, an important transcription factor that regulates antioxidant genes, have been developed and show promising therapeutic effects in multiple diseases. However, in diseases caused by complex pathogenic factors, antioxidant therapy is primarily approved to alleviate the toxic side effects of radiotherapy and chemotherapy or to be used as an adjunctive treatment rather than as a primary therapeutic approach. Furthermore, some studies have found that antioxidant therapy may exacerbate disease progression, which may be attributed to the fact that redox processes do not play a singular role in disease development; instead, they can influence disease progression through their role as second messengers, modulating multiple pathways via redox modifications. Consequently, simple antioxidant therapy may yield counterproductive effects in the treatment of complex diseases. Therefore, when formulating treatment strategies for complex diseases, it is essential to fully consider the complexity of redox regulation and the biological functions of downstream redox sensors.

With technological advances, numerous redox sensors have been detected and identified. However, due to the reversible and transient nature of redox signaling, dynamic monitoring and precise targeting remain significant challenges in current research. Currently, the analysis of the biological functions of reactive cysteine residues is still in its preliminary stages, lacking a systematic research framework, and there are relatively few comprehensive reviews focused on redox modifications. Therefore, systematically summarizing and promoting further research on the biological functions of redox sensors is of significant importance for a better understanding of the mechanisms by which redox regulation contributes to the occurrence and progression of diseases. There are already small molecule drugs targeting specific reactive cysteine residues in particular redox sensors that have shown promising therapeutic effects in preclinical studies. These precise targeting strategies can effectively reduce off-target effects and hold the potential for achieving precise redox regulation. However, relative to the vast array of redox-modified proteins, these studies remain insufficient. Additionally, screening small molecule drugs for each reactive cysteine residue site remains a time-consuming and labor-intensive endeavor. Therefore, the introduction of artificial intelligence-powered computer-aided drug design in this field may be an effective means to rapidly advance research and development.
